# Noninvasive Ultrasound Retinal Stimulation for Vision Restoration at High Spatiotemporal Resolution

**DOI:** 10.34133/2022/9829316

**Published:** 2022-02-21

**Authors:** Xuejun Qian, Gengxi Lu, Biju B. Thomas, Runze Li, Xiaoyang Chen, K. Kirk Shung, Mark Humayun, Qifa Zhou

**Affiliations:** ^1^Department of Biomedical Engineering, University of Southern California, Los Angeles, CA 90089, USA; ^2^Department of Ophthalmology, University of Southern California, Los Angeles, CA 90033, USA; ^3^USC Ginsburg Institute for Biomedical Therapeutics, University of Southern California, Los Angeles, CA 90033, USA

## Abstract

*Objective*. Retinal degeneration involving progressive deterioration and loss of function of photoreceptors is a major cause of permanent vision loss worldwide. Strategies to treat these incurable conditions incorporate retinal prostheses via electrically stimulating surviving retinal neurons with implanted devices in the eye, optogenetic therapy, and sonogenetic therapy. Existing challenges of these strategies include invasive manner, complex implantation surgeries, and risky gene therapy. *Methods and Results*. Here, we show that direct ultrasound stimulation on the retina can evoke neuron activities from the visual centers including the superior colliculus and the primary visual cortex (V1), in either normal-sighted or retinal degenerated blind rats *in vivo*. The neuron activities induced by the customized spherically focused 3.1 MHz ultrasound transducer have shown both good spatial resolution of 250 *μ*m and temporal resolution of 5 Hz in the rat visual centers. An additional customized 4.4 MHz helical transducer was further implemented to generate a static stimulation pattern of letter forms. *Conclusion*. Our findings demonstrate that ultrasound stimulation of the retina *in vivo* is a safe and effective approach with high spatiotemporal resolution, indicating a promising future of ultrasound stimulation as a novel and noninvasive visual prosthesis for translational applications in blind patients.

## 1. Introduction

Retinal degenerative diseases, caused by progressive degeneration of the light-sensitive photoreceptors in the retina, are one of the major causes of vision loss and blindness worldwide. Despite the loss of sensitivity to light, the remaining visual pathway is mostly intact and functional, allowing the visual prostheses to emerge as tools to restore vision. To be specific, microelectronic retinal prosthetics [1] was first proposed in 1956, which attempted to restore vision by bypassing the damaged photoreceptors and directly stimulating the inner retinal neurons. With the rapid development of electronic technology, some of these devices [2, 3] have been translated from the laboratory to the clinic and been successfully implanted in patients, such as Argus II device [4–6]. However, the challenges of the invasive implant of electronic devices, limited amounts of electrodes, considerable surgical costs, and potential surgery side effects have remained unsolved. To conquer these challenges, many efforts have been recently made, including the investigation of optogenetics [7, 8], near-infrared sensors [9], sonogenetics [10, 11], and gene therapy [12, 13]. Although favorable results have been reported, all these methods still require risky invasive procedures and comprehensive gene engineering. Therefore, an approach which can apply directly on the naturally existing mechanoreceptors to recover visual function in blind patients is desired.

Ultrasound (US) has been widely used in the clinic as a noninvasive approach for diagnostic imaging and therapeutic applications. Its ability to modulate the central nervous system has been shown since 1958. With the attractive features of noninvasiveness, deep penetration to cover the whole brain, and spatial selectivity on the order of submillimeters, US neuromodulation is a promising technology in the field of neuromodulation. Despite that the mechanism of US neuromodulation is still under investigation [14, 15], efforts have been made to exploit the potentials of US neuromodulation to treat various nerve-related diseases [16–18]. For the purpose of vision restoration, several pioneer studies have explored the feasibility of US stimulation of the retina to potentially evoke neuron activities [19–22]. However, the lack of *in vivo* demonstration of vision restoration and potential pattern generation (i.e., letter forms) from retinal degenerative models (i.e., blind animal cases) at a high spatiotemporal resolution impeded the role of US stimulation as an efficient vision restoration approach.

To move beyond the limitations of the previous US stimulation studies on the retina, we aim to demonstrate US as a promising approach to induce the neuron activities in the Royal College of Surgeons (RCS) rat *in vivo*, a retinal degenerative animal model widely used for assessing therapeutic effects. The most important contribution of our work is that it is the first time to observe that the US stimulation can reliably activate the degenerative retina *in vivo* with a high spatiotemporal resolution while light stimulation failed to show any retinal responses. We demonstrated that ultrasound stimulation of the retina can evoke neuron activities in the contralateral visual pathways including the superior colliculus (SC) and the visual cortex (VC) of the brain. Based on the retinotopic map properties of the SC [23], our study deciphered the static stimulation pattern of letter forms of US-induced visual activities in the retina. We further exploited the US-based noninvasive visual prosthesis as a potential vision restoration approach via the investigation of its standards of safety.

## 2. Results

**Figure 1:**
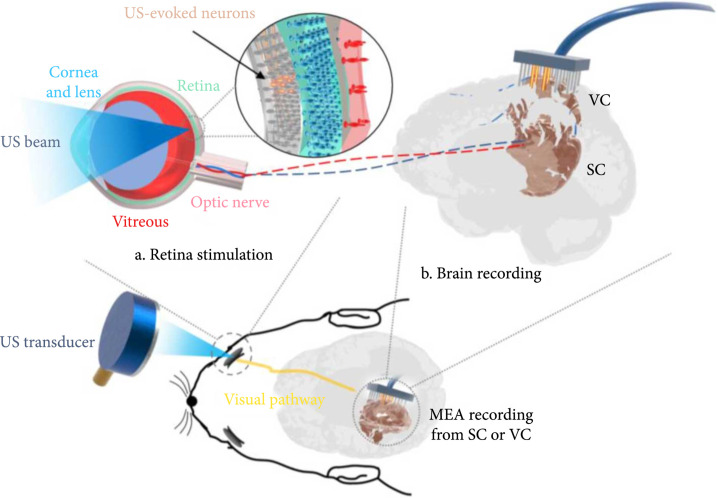
Schematic diagram of the US stimulation system. (a) The retina stimulation part. A 3.1 MHz spherically focused single-element transducer was used to transmit acoustic waves targeting at the retina. Retinal neurons were excited and then generated neural signals transmitting through the optic nerve to the brain. (b) The brain recording part. A multielectrode array (MEA) was inserted into the contralateral superior colliculus (SC) or visual cortex (VC).

A schematic overview of the US stimulation system is shown in Figure 1 where the implementation of the US sequence is defined in Supplementary Figure 1. The custom-build US transducer has a central frequency of 3.1 MHz with a focal depth of 10 mm. The characterization of the US transducer was performed by using a calibrated hydrophone as shown in Supplementary Figure 2. To be specific, the full-width-half-maximum (FWHM) beamwidth and the depth of focus (DOF) of the US transducer are 590 *μ*m and 4400 *μ*m, respectively. The free-field negative peak pressure (NPP) at the focal point, which is linearly related to the driving voltage of the US transducer, was measured to calculate the corresponding mechanical index (MI). To further investigate the effect of US attenuation and US-induced temperature change generated by the eye structure, both a finite element analysis (FEA) and an ex vivo experimental test were conducted in this study. As shown in Supplementary Figure 3, the FEA implied that the eye structure generated -2.0 dB attenuation while the attenuation of −3.3±0.4 dB was measured in the ex vivo test. These results were in line with our expectations since the backside sclera at *ex vivo* condition would contribute to a higher attenuation. In terms of US-induced temperature rising under the US parameters of 2.83 MPa NPP and 200 ms US duration, our ex vivo experimental measurement was consistent with our simulation study (which is 2°C in simulation and 1.8°C in the experiment).

**Figure 2:**
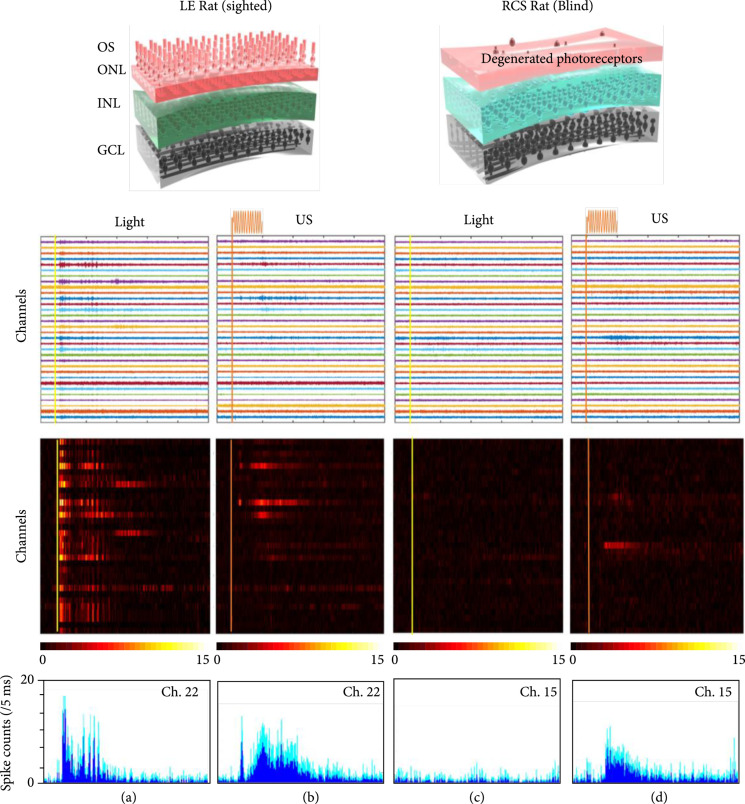
Examples of the evoked neuron activities recorded from SC *in vivo*. (a) Light response and (b) US response from one normal-sighted rat. (c) Light response and (d) US response from one RCS blind rat. The first row shows 500-7000 Hz bandpass-filtered signals with a scale of ±80 *μ*V in a recording time ranging from -0.1 s to 1 s. The second row shows the average spike counts per 5 ms of all channels. In the third row, a representative channel was randomly selected to demonstrate the spike count curve. The deep blue shows the average value while the baby blue shows the standard deviation (the data was plotted with 12 repeated trials).

Prior to conducting the US stimulation experiments, the vision of rats was evaluated using full-field light stimulation. For the purpose of electrophysiological recording from the visual centers, the contralateral area of the skull was removed by a partial craniotomy. The neuron activities evoked by the light stimuli were recorded using a 32-channel multichannel electrode array (MEA) placed either on the VC (V1 area) or inside the surface layer of the superficial SC (~150 *μ*m depth) after removing the overlying VC area. Our results in Figures 2(a) and 2(c) and Supplementary Figure 4(a, c) demonstrated robust visual activities to light stimulation in normal-sighted rats while in retinal degenerated RCS rats, no such light-evoked visual activities were noticed.

For the *in vivo* US stimulation experiments, the US transducer driven by a power-amplified sinusoid tone burst signal (see Methods) was placed in front of the rat eye and coupled with degassed gel. In both normal-sighted rats and RCS blind rats, prior to stimulating the eyes, the US stimulation beam was directed away from the eyeball to conduct control experiments to examine off-target effects. As expected, we did not observe any responses in such control experiments, indicating that there are no potential electrical or auditory confounds.

### 2.1. US-Evoked Neuron Activity in Normal-Sighted and RCS Blind Rats *In Vivo*

To demonstrate whether US stimulation is an efficient tool in evoking neuron activities of the rats *in vivo*, 10 normal-sighted LE rats (SC recordings were conducted in 8 rats, and VC recordings were conducted in 2 rats) were used in this study as listed in Supplementary Table 1. Examples of the US-evoked neuron activities recorded from the SC and VC of normal-sighted rats are shown in Figure 2(b) and Supplementary Figure 4(b), respectively. Unlike the light-evoked spike activities that constantly appeared after a short latency for most of the recording channels, the US-evoked spike activities were observed only in comparatively few channels. This is because that the light stimulator used in this study was a full-field strobe flash; the light stimulation is capable of activating the entire retina, resulting in neuron responses from almost all the recording channels. By contrast, the US-stimulated region of interest (ROI) determined by the focal zone of the US transducer is relatively small (at a few hundred microns level). As a consequence, as observed in Figure 2(b), only a few MEA channels that presumably corresponded to the US stimulated region of the retina showed spike activities. It should be noted that, despite the variation in the degree of US-evoked neuron activities between rats, all the normal-sighted rats showed neuron activities comparable to the responses during light stimulation.

Our results in normal-sighted rats indicated that US can be an alternative approach to stimulate the retina *in vivo*. To further validate the advantage of using US stimulation for vision restoration, a rat model of retinal degenerative blindness was investigated. The RCS rats that are considered to be totally blind after the age of 6 months were used in this study. To ensure a fair comparison with normal-sighted rats, the same US stimulation parameters (i.e., intensity and duration) were used in both study groups.

Based on the results from 16 RCS blind rats (14 rats for SC recording and 2 rats for VC recording), it is demonstrated that US is capable of stimulating the retinal neurons as evidenced by neural signals from the SC (Figure 2(d)) and VC (Supplementary Figure 4(d)). Based on the response amplitude and response duration, the US-induced neuron activities in blind rats were generally weak compared to the responses from normal-sighted rats.

### 2.2. The Effects of US Parameters on Neuron Activity

**Figure 3:**
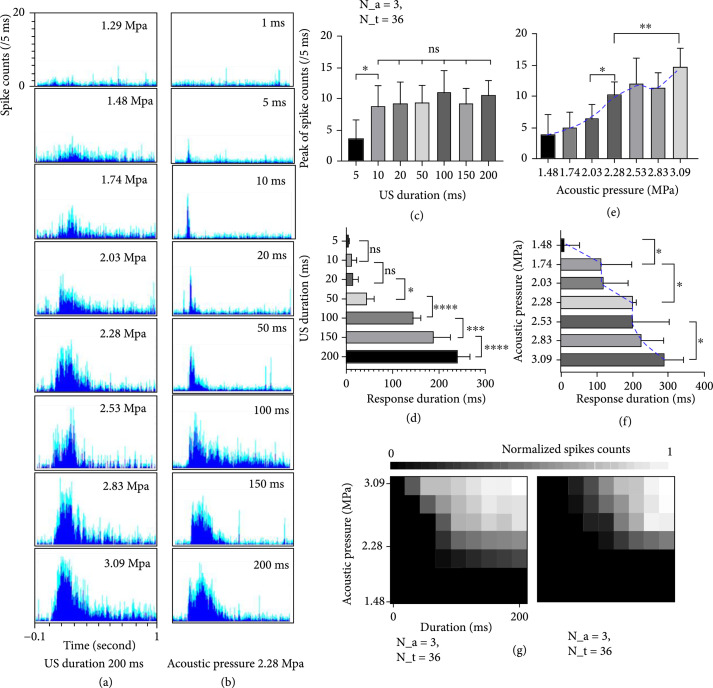
The neuron activities affected by US parameters. (a) Response changes along with different US intensities. (b) Response changes along with different US duration. (c) Statistical analysis of the relationship between US duration and response amplitude. (d) Statistical analysis of the relationship between US duration and response duration. (e) Statistical analysis of the relationship between US intensity and response amplitude. (f) Statistical analysis of the relationship between US intensity and response duration. (g) Response changes with US intensity and US duration: normal-sighted rats (left) and RCS blind rats (right). It should be noted that N_a represents the number of animals used in this analysis while N_t represents the repeated trials per each animal.

The influence of US intensity and US duration, the two major US stimulation parameters that showed substantial influence on evoked neuron activities, was further investigated. To conform with the US Food and Drug Administration (FDA) recommendation on maximal MI of 1.9, which corresponds to an NPP of 3.35 MPa at 3.1 MHz, we varied the driving voltage of the US transducer from 200 mV (which is 1.29 MPa in NPP) to 600 mV (which is 3.37 MPa in NPP) with an interval of 50 mV. Figure 3(a) shows the neural response under various US intensities. In addition, the US duration from 1 ms to 200 ms was explored, in which the results were shown in Figure 3(b). Figures 3(c)–3(f) list the statistical comparisons among various US intensities and US duration on evoking neuron activities. All recordings were repeated 12 times for statistical analysis.

The quantifications of neuron activities were characterized by the response amplitude and the response duration (see Methods for detailed description), respectively. It was observed that the response amplitude did not vary with 10 ms or longer US duration, but the response duration increased with the increase in US duration (Figure 3(d)). With respect to the increasing US intensity, both response amplitude and response duration were increased (Figures 3(e) and 3(f)). In summary, the overall acoustic energy (which is the combination of US intensity and US duration) determined the threshold of evoking neuron activities. It was also found that the blind RCS rats have a higher stimulation threshold level compared to the normal-sighted rats (Figure 3(g)).

In addition, we investigated the performance of US stimulation on the basis of two sequence modes, including continuous tone burst mode and pulse mode with duty cycle. As indicated in Supplementary Figure 5, both of these modes have the capability to stimulate the retina *in vivo*. Under the condition of the same time-averaged (that is, duty cycle×US duration) US sequence, there is no statistical difference in the total number of spikes. In particular, a higher duty cycle was able to generate larger response amplitude while a lower duty cycle tended to maintain longer response duration (Supplementary Figure 5(b)). These results implied that the US stimulation sequence has the potential to determine the response features.

### 2.3. Spatiotemporal Resolution of US-Evoked Neuron Activity

**Figure 4:**
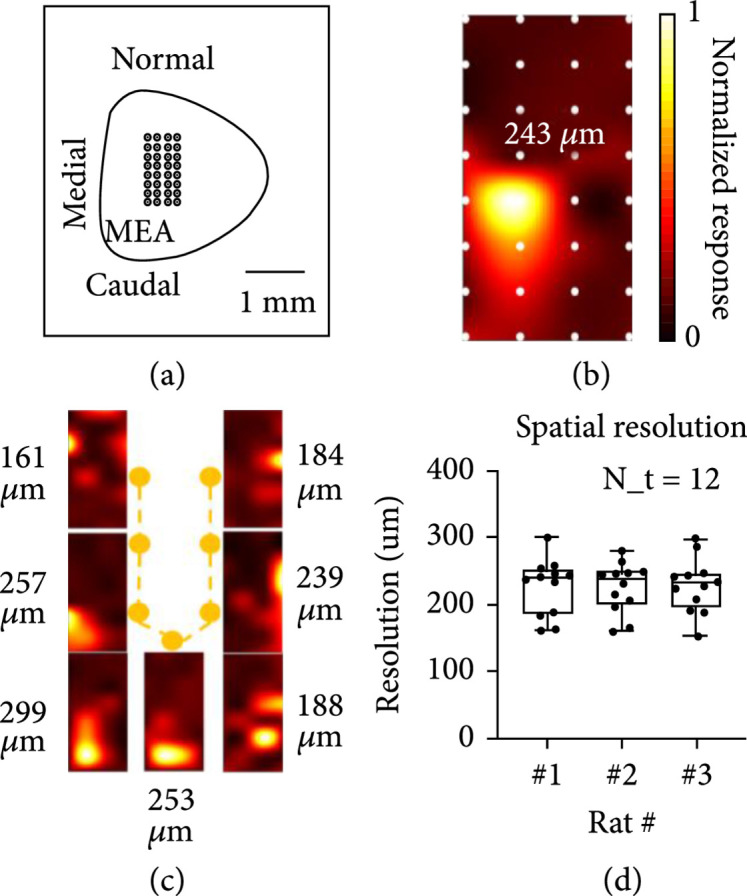
The spatial resolution of ultrasound stimulation. (a) Schematic diagram of the 32-channel MEA placed on the surface of SC. (b) One representative US stimulated response mapping where the distance between each adjacent electrode (white dot) is 150 *μ*m. (c) Different positions of SC were activated by controlling US-stimulated retinal regions via moving the spherically focused transducer manually. (d) Spatial resolution summary over 3 RCS blind rats. N_t is the repeated trials per each rat.

We further explored the spatial and temporal resolution of the retinal US stimulation in the SC. As regards the spatial resolution, we reconstructed the distribution of US-evoked neural responses across the SC surface based on the response amplitude without changing the position of the US transducer (Figure 4(b)). Then, we manually changed the positions of the US transducer using the 5-axis precision stage to validate the ability to activate different regions of the SC by shifting the focal point of US beam. As shown in Figure 4(c), the response pattern in the form of letter “U” was observed in the SC, indicating that the US-stimulated ROI of the retina activated the corresponding SC region. The activated SC regions varied with spatial resolution (defined as the averaged FWHM of medial and caudal directions) ranging from 161 *μ*m to 299 *μ*m. Such variation in spatial resolution could be attributed to the curvature of the retina since the US-induced retinal region could be different from the US beam pattern measured at the free-space domain. Despite these variations, our US system still achieved a good spatial resolution at an average level of 250 *μ*m on resolving the response patterns.

**Figure 5:**
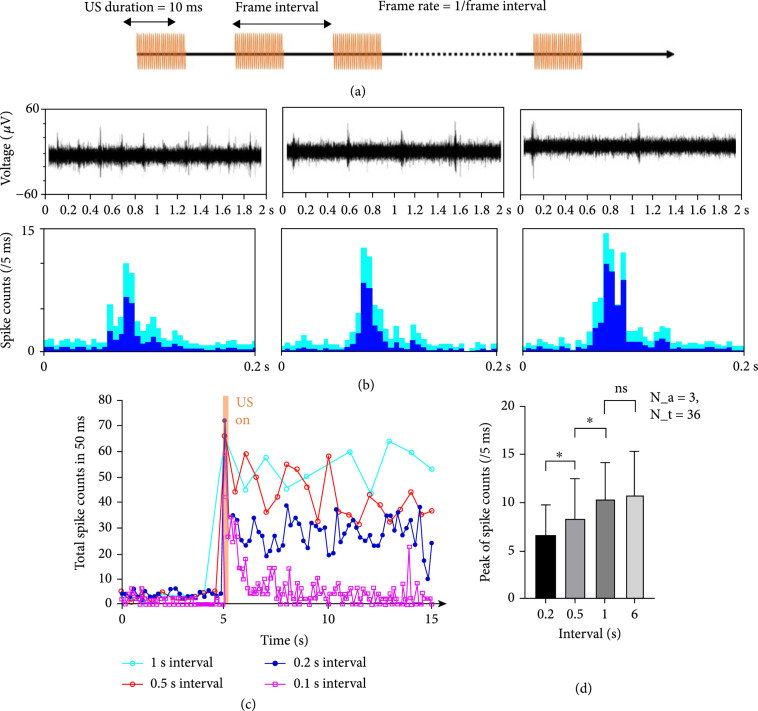
The temporal resolution of ultrasound stimulation. (a) Schematic diagram of the implemented US sequence. (b) The filtered raw signals and spike counts of the US response with the temporal resolution of 5 Hz, 2 Hz, and 1 Hz (from left to right). (c) The total spike counts when the US stimulation switched from off to on. US response remained stable until reaching the frame rate of 10 Hz. (d) The statistical comparisons among various frame rates. It should be noted that N_a represents the number of animals used in this analysis while N_t represents the repeated trials per each animal.

The temporal resolution of US stimulation (namely, frame rate) which was defined as the minimal frame interval capable of evoking stable neuron activities is another important factor that can influence the functionality of a potential visual prosthesis. This factor could determine whether the visual prosthesis is able to provide a smooth vision for dynamic objects or not. In this study, we conducted a consecutive 20-second US stimulations with different frame rates under the US parameter settings of 10 ms US duration and the 2.83 MPa NPP. As shown in Figure 5(b), representative 2-second signals were randomly cropped from the 20-second raw data. Our results indicated that stable neuron activities were achieved up to the frame rate of 5 Hz (see Figure 5(c)), while a higher frame rate (i.e., 10 Hz) could potentially suppress the firing neurons in a short time (<5 seconds, yellow line in Figure 5(c)). Our results were consistent with the previous electrical stimulation experiments conducted in rats [24], which showed that suppression of the evoked responses in the SC was observed with increasing stimulus frequency.

To validate that such suppression at higher frame rates was caused by neuron saturation rather than the neuron damage, we performed an additional stimulation study at a lower frame rate to the same ROI right at the end of the previous high frame rate stimulation. As a result, the similar levels of neuron responses (response amplitude and response duration) were observed again, indicating that US stimulation at a high frame rate is safe.

### 2.4. Static Stimulation Pattern Induced by the Helical Transducer

**Figure 6:**
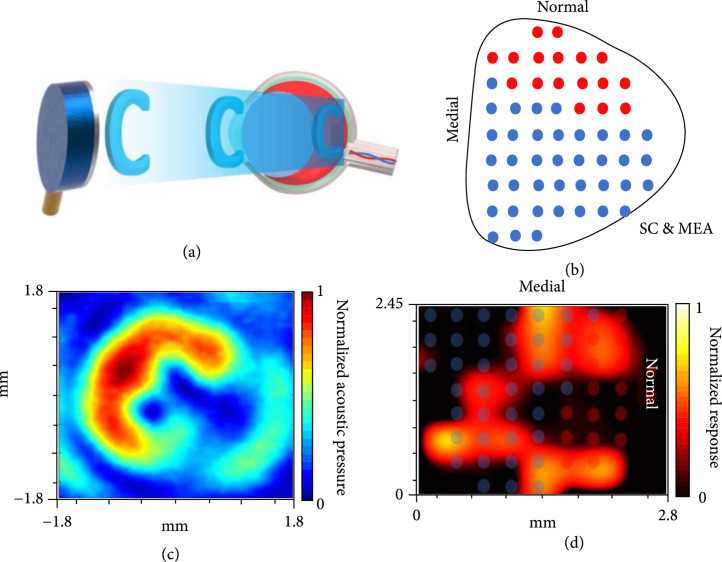
The static pattern generation of the letter form “C” using a customized helical US transducer. (a) Schematic diagram of generating a beam pattern of “C” to the retina *in vivo*. (b) The 56-channel MEA placed on the whole surface of SC. (c) The hydrophone-measured acoustic field with a pattern “C” at focus. (d) The MEA recorded US-evoked neuron activities at SC.

Owing to the single-point beam shape of the spherically focused US transducer, we designed and fabricated a novel helical transducer in order to generate a specific beam pattern of the letter form “C” (Figure 6(a)). Different from the previously used 32-channel MEA with a 150 *μ*m spacing to precisely calculate the spatial resolution of the US-evoked neuron activities, herein we designed and implemented a 56-channel MEA with a 350 *μ*m spacing (Figure 6(b) and Supplementary Figure 6) to cover the whole SC region for better visualization of the letter form “C”. As shown in Figure 6, our observed response pattern of US-evoked neuron activities (Figure 6(d)) was consistent with the measured hydrophone results at the water condition (Figure 6(c)), indicating that static US stimulation is able to create the same pattern of neuron activities in SC.

## 3. Discussion

In the past years, significant attempts were made to restore functional vision in blind patients using visual prostheses. Although several types of visual prostheses including retinal [25, 26], optic nerve [27], and cortical prostheses [28] have been tested, retinal implants attracted the most attention since it can function based on natural information processing along the visual pathway. However, the majority of these devices require invasive surgery to implant the device [29, 30]. In addition, current technologies of electrical stimulation have limited spatial resolution due to the inherent difficulty of complex implantation procedures of a large number of electrodes. Although recent studies have explored other neuromodulation technologies such as optogenetics and sonogenetics via gene therapy strategies, all these approaches still require complex and risky gene engineering.

As an innovative strategy, we successfully demonstrated that our extraocular US stimulation system is capable of eliciting the neuron activities in a rat model of degenerative blindness. As a noninvasive, safe, and cost-effective technology, direct US retinal stimulation showed great promise for future translational application. We report here the feasibility of using the US technique to stimulate and generate response patterns at a spatial resolution level comparable to that of the first FDA-approved retinal prosthesis—Argus II [21]. In addition, our study demonstrated that the US stimulation technique can achieve good temporal resolution for the smooth transfer of the visual information and ruled out the safety concerns for the implementation of US technique as a potential retinal prosthesis. To validate the capability of visual pattern generation, we further designed a helical US transducer and successfully demonstrated letter “C” shape pattern by static US stimulation.

Owing to the fact that visual information is preprocessed in subcortical relay centers such as the SC (the rat SC receives projections from 85% to 90% of the retinal ganglion cells from the contralateral eye) and is finally processed in the VC, our experiments were designed to record neuron activities from both SC and VC. To establish the baseline of US stimulation, our study was first conducted in normal-sighted rats having healthy and functional photoreceptors to generate bioelectrical signals under natural light stimulation. As expected, we observed robust light-evoked neuron activities in both SC and VC. Comparable neural activity was also elicited during US stimulation suggesting that US could be used as an alternative approach to generate encoded visual sequences.

To further illustrate the advantages of using US stimulation for visual restoration in blind subjects, the *in vivo* effectiveness of US stimulation was investigated in a rat model of degenerative blindness (RCS above 6 months of age). With the loss of functional photoreceptors, light stimulation failed to evoke neuron activities in both SC and VC of RCS rats. By contrast, US stimulation was able to stimulate the retinal neurons, to evoke spike activities in the higher visual centers of the brain of blind RCS rats. Our study is the first *in vivo* demonstration of US-based restoration of retinal function in blind rats. Although the detailed functional mechanism of US-induced stimulation activities in the retina is not yet fully understood, the “proof of concept” for using US as a noninvasive retinal prosthesis for clinical application to treat blindness is demonstrated.

For future translational applications, it is important that the potential bioeffects (i.e., thermal effect and cavitation effect) of the US stimulation should be considered to ensure safety. Owing to the pilot study, there is currently no standard regulation that could be followed or compared. We consider that our US stimulation approach is safe on the basis of the following two aspects. First, we calculated the MI under all US parameters we used. Despite that the MI in our current study was relatively high (i.e., ensuring greater efficiency of stimulation such as consistent responses and good signal-to-noise ratio) compared with the FDA maximum MI requirement of 0.23 for ophthalmological use, the US intensity and MI were within a reasonably safe range compared with former US stimulation studies [20, 22]. In other words, the US intensity can be further reduced to enhance the safety of US stimulation technology. Second, to assess whether US stimulation can damage retinal tissue, we collected histology sections of the retina from the stimulated eyes (see Supplementary Figure 7). Representative histology results of US-stimulated retinas from both normal-sighted rat and retina degenerative rats were examined. The data suggested that the retina structure remain intact even after 2 hours of US stimulation. No obvious changes in the retinal architecture were noticed confirming the absence of damage to any of the retinal layers due to US stimulation.

We further investigated the influence of changes in the US sequence on evoked neuron activities. We observed that, by controlling the time-averaged US intensity and US duration, the response amplitude and response duration can be modified. Our results demonstrated that continuous ultrasound waves and pulsed ultrasound waves with various pulse repetition frequencies (PRF) can generate similar neuron activities on the basis of the same average power level. Such an observation (i.e., only average power is important) is in agreement with *in vitro* retinal studies by Menz et al. [22]. However, there are other studies reporting that pulsed ultrasound with a PRF of 0.5-2 kHz range is more effective than continuous wave in evoking neuron activities [31]. In particular, Kubanek et al. [32] indicated that the behavior response of *Caenorhabditis elegans* to ultrasound stimulation depended on the PRF and duty cycle. They claimed that the ideal parameters were 1 kHz PRF and 50% duty cycle. The above inconsistencies in these studies could be attributed to the species difference and associated differences in the types of neurons that were stimulated. It has been reported that certain types of neuron cells or mechanosensitive ion channels (i.e., the auditory nerve fibers and hair cells) are selectively sensitive to the stimulation with specific frequency range [33]. Overall, the optimal PRF and duty cycle for US stimulation on the retina remain as an open question, which will be the future direction of our work. Another important factor related to the US sequence is the temporal resolution of US-evoked neuron activities. We demonstrated that the temporal resolution could be increased when a more effective US retinal stimulation sequence is deployed. Although our results have only shown a frame rate of 5 Hz, higher frame rates are expected to be achievable by using less powerful US.

The previous literature has indicated that pulsed US stimulation could potentially activate auditory pathways [34] and the corresponding responses could further spread to other cortical areas [35]. It was also reported that patients with earplugs heard tones in the same frequency of Doppler ultrasound’s repetitive frequency [36]. To eliminate the potential auditory confounds, during our pilot US stimulation experiments, the stimulation was initiated from the area of the auditory pathway. As expected, no stimulation-induced responses were observed in the above control experiments. Moreover, SC mapping data (Figure 4) demonstrated that the neuron activities are confined to a small SC area presumably corresponding to the focal retina area that was stimulated. Therefore, it is confirmed that the neuron activities recorded from the SC and VC are the neural signals generated by the US-stimulated retina region.

The phenomena of delayed response and lower response amplitude observed in human patients are considered the hallmarks of retinal degeneration diseases [37–41]. Delayed response onset latency and reduced response amplitude during light stimulation were observed in various retinal degenerative rat models [42, 43]. In the present study, it was noted that, even in normal-sighted rats (Supplementary Figure 8), the response onset latency for light stimulation (20.18+3.18 ms) was significantly shorter compared to US stimulation responses (43.88+9.55 ms, P<0.001). Based on the previous light stimulation experiments conducted in normal rats, a direct correlation between the stimulus intensity and response onset latency was observed [44]. The shorter response onset latency observed at higher light stimulation (0.81 log cd/m^2^) was suggested to be due to the more rapid processing of the photic signals. According to these observations, an overall delay in the retina response during US stimulation can be related to the decreased stimulation effects. Other possible explanations for the delayed response include a slower response by the US-sensitive neurons compared to the light-driven pathways, or US-sensitive neurons require time to accumulate energy before reaching the threshold for activation. Another interesting finding of US stimulation is that the response onset latency of blind rats (86.68+18.98 ms) was significantly higher than that of normal-sighted rats (P<0.001). This can be attributed to the overall changes in the retinal homeostasis associated with prolonged visual deprivation or due to the remodeling taking place in the RCS retina during advanced disease conditions [45].

The current investigation on US neuromodulation mechanism relies in two aspects, namely, physical mechanism and neuronal mechanism. In terms of the neuronal mechanism, many studies have investigated the role of various mechanosensitive ion channels in US stimulation [46–50]; however, their conclusions are inconsistent and inconclusive. A more comprehensive study is still required to uncover the neuronal mechanism of US stimulation of the retina. Regarding the physical mechanism, acoustic radiation force (ARF), cavitation, and thermal effect are the major contributing factors. In particular, our US-induced temperature increase is less than 2°C at water condition (testing US parameters: 2.83 MPa NPP, 200 ms duration). On the basis that the temperature increase during *in vivo* condition should be even lower due to the blood flow perfusion, it is unlikely to evoke temperature-sensitive neurons [51]. With respect to the cavitation effect, all MIs in our study were lower than the FDA-required threshold of 1.9 for diagnostic US imaging. Therefore, the cavitation effect could be neglected herein. As a consequence, ARF is considered to be dominant of the physical mechanism of US stimulation, which is supported by both our experimental results and previous studies [20] based on ex vivo retina stimulation study. ARF is a nonlinear effect that transfers the momentum from propagating waves to tissues. Specifically, ARF whose magnitude is determined by the temporal average intensity of the US and the absorption coefficient of the tissue (ARF=2αI/c, where I is the intensity, c is the speed of sound in the medium, and α is the absorption coefficient) will lead to a mechanical displacement of tissue. Such a displacement could stretch or compress the cell membrane and cause the changes in membrane capacitance or the activation of mechanosensitive ion channels.

The next step of our study would be to use a 2D matrix array for retinal stimulation. In comparison to the single-element US transducer with a fixed beam pattern (that is, single point for spherically focused transducer and letter “C” for the helical transducer) at a certain ROI, the implementation of the 2D array has the following advantages. First, the 2D array with the imaging capability could provide the potential guidance to precisely alter the focal zone along the retina with curvature, which maybe also benefits to reduce the US intensity level on evoking neuron activities. Second, the 2D array with the ability to electronically steer the beam in a 3D domain would be helpful in pattern generation such as arbitrary and dynamic patterns on the retina, which is practically useful in vision restoration. Finally, the anatomy of the eyeball, especially the cornea and lens in the anterior eye, could potentially affect the accuracy of the beam pattern generated in the single-element US transducer. In other words, the reflection and refraction of US waves caused by these parts can distort the designed beam pattern. However, the array’s ability to independently control the amplitude and phase of each element can compensate for the distortion using inversion algorithms [52, 53].

Lastly, it is important to note that either the 3.1 MHz or 4.4 MHz transducer was primarily chosen in this study based on two aspects. One is the compromise among *in vivo* feasibility, good spatial resolution, and the capability of sustaining high US intensity and long US duration; the other is that the center frequency of the US transducer ranging from 2 to 10 MHz could provide spatial resolution similar to that of the first FDA approved retinal prosthesis—Argus II [21]. It has been well established that higher US frequency is associated with better spatial resolution, resulting in a more precise manner for retinal stimulation. However, the significant US attenuation and its potential thermal effect are unavoidable at higher US frequency, especially when the cornea and lens tissue are presented for *in vivo* study [54]. Therefore, the selection of optimal US frequency with respect to the proper trade-off between US attenuation and spatial resolution is our ongoing work.

In conclusion, these results represent a step towards noninvasive retinal prosthesis development using US. The *in vivo* demonstration of visual restoration in blind rats suggested that US opens a new avenue for the development of a novel noninvasive retinal prosthesis. Unlike light-evoked responses, the US stimulation may show different response patterns that can be modulated by US intensity and US duration.

## 4. Methods

### 4.1. Experimental Setup

The schematic diagram of the experimental setup with US stimulation is shown in Figure 1. A dual-channel function generator (AFG3252C, Tektronix, Beaverton, OR, USA) was implemented in this study to control the stimulus sequence and trigger signals for data acquisition. More specifically, the output of channel 1 was used to generate the stimulus sequence, followed by an RF power amplifier (100A250A, Amplifier Research, Souderton, PA, USA) with a gain of 50 dB, and then used to drive a custom-build US transducer. Channel 2 which is self-synchronized with channel 1 through the internal system clock will send synchronized trigger signals to the interface board of an electrode recording system—Lablynx (Neuralynx, Bozeman, MT, USA). Regarding the light stimulation, a full-field strobe flash using a Grass photic stimulator (Grass Instrument Co., W. Warwick, RI, USA) was delivered to the contralateral eye; in the meanwhile, the stimulator sends out a trigger signal to the Lablynx recording system for data recording. The time interval between each adjacent trigger signal is set to 6 seconds in order to ensure the evoked potential activities back to normal.

To record multiunit neuron activities (MUA) from the SC or VC, two microelectrode arrays (MEA, Microprobes for Life Science, Gaithersburg, MD, USA) with an impedance of 0.5 mega-Ohms were used in this study. Specifically, the 32-channel (4 by 8) MEA with a finer spacing (150 *μ*m) between adjacent electrodes was used to measure the spatial resolution of the US-evoked neuron activities. The 56-channel MEA with a spacing of 350 *μ*m (Supplementary Figure 6) has a larger recording area and thus can cover the whole surface of the SC. Such a design was used to record the US-evoked response pattern from SC. Signals from MEA were sampled at 30 kHz by the analog-to-digital multiplexing headstage (HS-32-MUX-PTB, Neuralynx) before transferring to the Lablynx recording system. The US transducer and the recording system were grounded together to minimize the artifacts.

### 4.2. US Parameters and Sequence for Stimulation

Supplementary Figure 1 shows the US parameters and the US sequence used in this study. Unless specified otherwise, the default US sequences used in this study were continuous waves with 100% duty cycle and a frame interval of 6 seconds. A 6-second interval was chosen to ensure the stimulated neurons fully recovered after each stimulation. It should be pointed out that the frame interval was only changed in the study of temporal resolution of US-evoked neuron activities.

The relationships between acoustic pressure (which is NPP in this study) and other US parameters such as MI, spatial peak pulse average intensity ($I_{\text{SPPA}}$), and spatial peak temporal average intensity ($I_{\text{SPTA}}$) are listed here: $\text{MI}=\text{NPP}\;\left(\text{MPa}\right)/\sqrt{f\left(\text{MHz}\right)}$, $I_{\text{SPPA}}={\text{NPP}}^{2}/2\rho c$, and $I_{\text{SPTA}}=I_{\text{SPPA}}\times \text{Duty}\;\text{cycle}$, where ρ and c are the density and sound speed in the medium.

To investigate the effect of US stimulation parameters on the evoked neuron activities, two stimulation parameters (US intensity and US duration) were explored. In terms of US intensity, we changed the driving voltage (which is the output voltage of the function generator before being fed into the power amplifier) from 50 mV to 500 mV with an interval of 50 mV. With respect to US duration, a test ranging from 50 ms to 200 ms with an interval of 50 ms was implemented.

To compare the continuous tone burst mode with pulse mode, we designed four different stimulation sequences, including one for continuous tone burst mode and three for pulse mode with 30%, 50%, and 70% duty cycle, respectively.

### 4.3. Animal Preparation

The *in vivo* rat experiments were performed according to the University of Southern California Institutional Animal Care and Use Committee (IACUC) protocol. A total of 26 rats (male and around six months old) were investigated, including 10 normally sighted Long-Evan (LE) rats and 16 RD Royal College of Surgeon (RCS) blind rats. Supplementary Table 1 lists the number of rats used in each subset of our study. The RCS rats are characterized by retinal pigment epithelium (RPE) dysfunction owing to the deletion of the Mer tyrosine kinase (MerTK) receptor that abolishes internalization of photoreceptor outer segments by RPE cells. For each rat, only one eye was used for stimulation purpose while the other eye was untreated.

Before the experiment, the rats were anaesthetized with an intraperitoneal injection of ketamine/xylazine (50-90 mg/kg, 5-10 mg/kg) and maintained under sevoflurane inhalation through a nose cone. For electrophysiological recording of the brain visual centers, a small craniotomy was made based on standard rat stereotactic coordinates to expose the VC. For the SC recording, the overlying VC was removed to expose the SC surface. The MEA was advanced into the VC and SC using the stereotactic apparatus. To ensure the sensitivity of the retina to light, all procedures were performed in a dark room illuminated with dim red light. During the experiment, the rat eye was first stimulated with light to establish the baseline of the retinal response, and then, the degassed ultrasound gel was used to couple the space between transducer surface and rat eye and finally tested with ultrasound stimulation to investigate its potential benefits. After the experiment, the rats were sacrificed and both eyes (i.e., the stimulated eye and the untreated eye) were kept for histology analysis in order to investigate the safety issue of our ultrasound stimulation sequence.

### 4.4. Ultrasound Transducer

Considering the size and the potential US attenuation of the eye, we have designed and fabricated a 3.1 MHz transducer with a focal length of 10 mm and the f-number of 1. The DL-47 (Del-Piezo Specialties, FL, USA) material was used as the piezoelectric layer due to its high-power sustaining capability. A layer of 10 *μ*m parylene was coated on the surface of the transducer for protection and insulation. During the experiment, the transducer was mounted on a 5-axis precision stage in order to accurately control the position of the transducer. The acoustic fields of the transducer were calibrated using a hydrophone (HGL-0085, ONDA Co., Sunnyvale, CA, USA). After obtaining the raw acoustic pressure signal data, we calculated the NPP, MI, and spatial peak pulse average intensity ($I_{\text{SPPA}}$) (Supplementary Table 2).

With respect to the pattern generation experiment, a single-element helical transducer with a center frequency of 4.4 MHz was designed and fabricated. It was made of 1-3 composite DL-48 (Del-Piezo Specialties, FL, USA) with the designed parameters of 4 mm outer diameter, 2 mm inner diameter, 10 mm focal length, and 1.2 mm vertical separation. The detailed information of the design and fabrication process of helical transducers can be found in our previous work [55].

### 4.5. Finite Element Analysis Simulation

The finite element analysis (FEA) simulation was conducted by using COMSOL Multiphysics 5.3a (Stockholm, Sweden). An acoustic module and bioheat transfer module were used in this study. In the simulation setup, the eyeball was simplified to four main parts: cornea, lens, vitreous body, and retina where the shape and size of each part were preset [56, 57]. The acoustic and thermodynamic properties of each part were set based on the previous literature [54]. Detailed parameters are listed in Supplementary Table 3.

### 4.6. US Aberration and US-Induced Heating in Ex Vivo Rat Eyeball

Two rat eyeballs were used to estimate the US attenuation caused by eye structure and the US-induced temperature increase. As regards the US attenuation measurement, the hydrophone was placed at the focal point of the transducer (10 mm in depth). The intact eyeball was held by a clip and placed on the top of the hydrophone with a gap about 1 mm. The acoustic pressure was repeatedly measured three times with and without the presence of the eyeball, respectively. Regarding the temperature measurement, a T-type wire thermocouple (XC-T-TC-WIRE, Omega Engineering Inc., CT, USA) was inserted into the posterior eye, and then, the temperature was read out from a thermocouple meter (RH820U, Omega Engineering Inc., CT, USA).

### 4.7. Data Postprocessing

The raw electrode signals were sampled at 30 kHz and stored for postprocessing in MATLAB 2019b (MathWorks). To obtain neuron spikes, a 500-7000 Hz bandpass filter was applied to SC data while VC data were filtered with a 300-7000 Hz bandpass filter. For SC and VC recording, a subpopulation of MEA channels that had strong background noise (presumably due to blood vessels) was manually deleted and excluded from final data analysis.

For each channel of the MEA, the maximal peaks with amplitude three times stronger than the background noise of this channel are considered spikes. The response was considered to be observed when the average spike counts per 5 ms was larger than 2. Response amplitude was defined as the peak of averaged spike counts per 5 ms. The response duration was defined by the time slot where the average spike counts per 5 ms was continuously larger than 2.

The total average spike counts in 500 ms of all channels over a time window of 500 ms were used to map the US-induced response distribution. In order to reconstruct the US-evoked response mapping at SC, 4 times modified Akima cubic 2D interpolation was conducted. Since the response regions have irregular shapes, the spatial resolution of US-evoked neuron response was determined as the averaged FWHM in the medial and caudal directions.

### 4.8. Histology Analysis

After the completion of the retina stimulation experiments, the rats were euthanized and eyes were either immersed in Bouin’s fixative or embedded in paraffin. Transverse sections of the retina were cut, mounted onto slides, and stained with hematoxylin-eosin (H&E). A series of sections through the full extent of each transplant was evaluated at the light microscopic level.

### 4.9. Statistical Analysis

Statistical significances between three or more were tested using ordinary one-way ANOVA and Tukey’s multiple comparison test. Prism 9 software (GraphPad) was used to calculate the values. Significance values are P<0.05 (∗), P<0.01 (∗∗), P<0.001 (∗∗∗), and P<0.0001 (∗∗∗∗).

## Data Availability

The authors declare that all data supporting the results in this study are available within the paper and its supplementary information. The raw and analyzed datasets generated during the study are available for research purposes from the corresponding author on reasonable request.

## Supplementary Materials

Supplementary Materials Figure S1: the schematic diagram of the US sequence and the definition of US parameters in our study. Figure S2: free-space US field and pressure measured in the hydrophone test. Figure S3: simulated results of US distortions and attenuation caused by the eyeball. Figure S4: examples of US-evoked neuron activities recorded from VC. Figure S5: the US stimulation response determined by duty cycle. Figure S6: the helical transducer for pattern generation of the letter form “C”. Figure S7: representative histology results. Figure S8: differences in the response latencies from both stimulation methods and both rat strains. Table S1: the number of rats used in each subset of our study. Table S2: the relationship between the driving voltage of the US transducer and acoustic parameters. Table S3: list of acoustic and thermal parameters of water and ocular tissue components.
