# Supplementary material for: Noninvasive Ultrasound Retinal Stimulation for Vision Restoration at High Spatiotemporal Resolution
Source: BME Front. 2022 Feb 21;2022:9829316. doi: 10.34133/2022/9829316 (PMC10521738; doi:10.34133/2022/9829316)
Supplement: Supplementary Materials — Figure S1: the schematic diagram of the US sequence and the definition of US parameters in our study. Figure S2: free-space US field and pressure measured in the hydrophone test. Figure S3: simulated results of US distortions and attenuation caused by the eyeball. Figure S4: examples of US-evoked neuron activities recorded from VC. Figure S5: the US stimulation response determined by duty cycle. Figure S6: the helical transducer for pattern generation of the letter form “C”. Figure S7: representative histology results. Figure S8: differences in the response latencies from both stimulation methods and both rat strains. Table S1: the number of rats used in each subset of our study. Table S2: the relationship between the driving voltage of the US transducer and acoustic parameters. Table S3: list of acoustic and thermal parameters of water and ocular tissue components. [file 9829316.f1.zip › Supplementary Information_revision.docx]

**Non-invasive ultrasound retinal stimulation for vision restoration at high spatiotemporal resolution**

Xuejun Qian^1,2,&^, Gengxi Lu^1,2,&^, Biju B. Thomas^2^, Runze Li^1,2^, Xiaoyang Chen^1^, K Kirk Shung^1^, Mark Humayun^1,2,3^, Qifa Zhou^1,2*^

1. Department of Biomedical Engineering, University of Southern California, Los Angeles, CA 90089, USA
2. Department of Ophthalmology, University of Southern California, Los Angeles, CA 90033, USA
3. USC Ginsburg Institute for Biomedical Therapeutics, University of Southern California, Los Angeles, CA 90033, USA

^&^These authors contributed equally

^*^Corresponding Author

**Supplementary Information**

|  | **SC recording** | | | | **VC recording** | **Total** |
| --- | --- | --- | --- | --- | --- | --- |
|  | **Base** | **Spatial resolution** | **Temporal resolution** | **Change US parameters** |  |  |
| **Normal sighted rats** | **3** | **0** | **0** | **5** | **2** | **10** |
| **RCS blind rats** | **3** | **3** | **3** | **5** | **2** | **16** |

**Supplementary Table 1: The number of rats used in each subset of our study.** ‘Base’ represents that the rat was performed with light stimulation followed by ultrasound stimulation.

| Input Voltage (mV) | Pressure (MPa) | Isppa(W/cm^2) | MI |
| --- | --- | --- | --- |
| 50 | 0.432 | 6.220 | 0.245 |
| 70 | 0.535 | 9.535 | 0.304 |
| 100 | 0.699 | 16.306 | 0.397 |
| 150 | 0.943 | 29.639 | 0.536 |
| 200 | 1.289 | 55.416 | 0.732 |
| 250 | 1.481 | 73.121 | 0.841 |
| 300 | 1.742 | 101.144 | 0.989 |
| 350 | 2.030 | 137.353 | 1.153 |
| 400 | 2.283 | 173.790 | 1.297 |
| 450 | 2.530 | 213.398 | 1.437 |
| 500 | 2.825 | 265.979 | 1.604 |
| 550 | 3.086 | 317.368 | 1.753 |
| 600 | 3.374 | 379.374 | 1.916 |

**Supplementary Table 2:** The relationship between the driving voltage of US transducer (before 50 dB gain via power amplifier) and acoustic parameters such as negative peak pressure, intensity and mechanical index.

|  | Density (kg/m3) | Sound speed (m/s) | Heat capacity at constant pressure (J/kg/K) | Thermal conductivity (W/m/K) | Attenuation (dB/cm/MHz) |
| --- | --- | --- | --- | --- | --- |
| Water | 1000 | 1500 | 4178 | 0.62 | 0 |
| Cornea | 1062 | 1586 | 4178 | 0.58 | 0.78 |
| Vitreous | 1005 | 1532 | 3999 | 0.6 | 0.01 |
| Lens | 1076 | 1647 | 3000 | 0.40 | 1.19 |
| Retina | 1034 | 1538 | 3680 | 0.57 | 1.15 |

**Supplementary Table 3:** List of acoustic and thermal parameters of water and ocular tissue components.

**
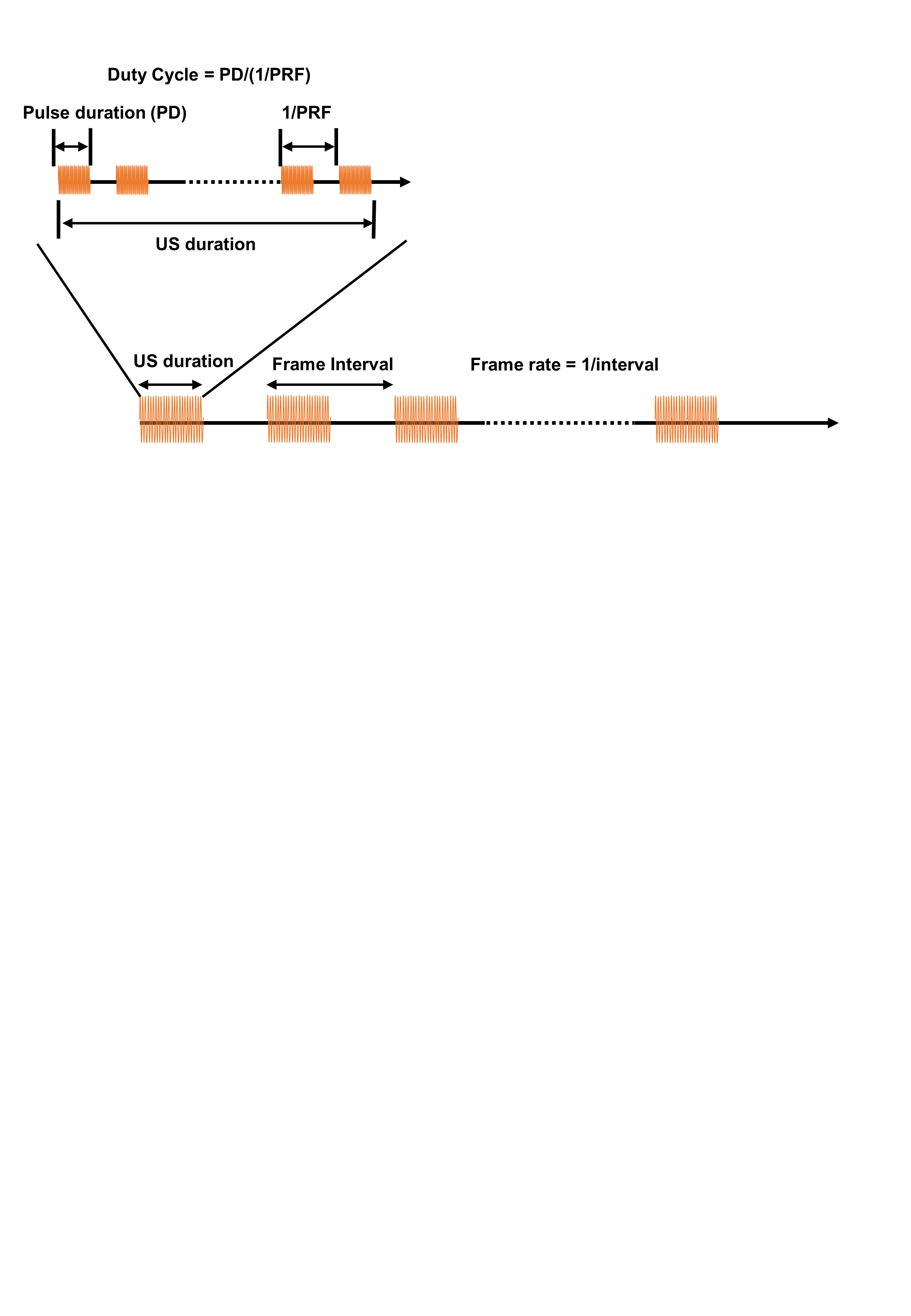
**

**Supplementary Figure 1:** The schematic diagram of the US sequence and the definition of US parameters in our study.

**
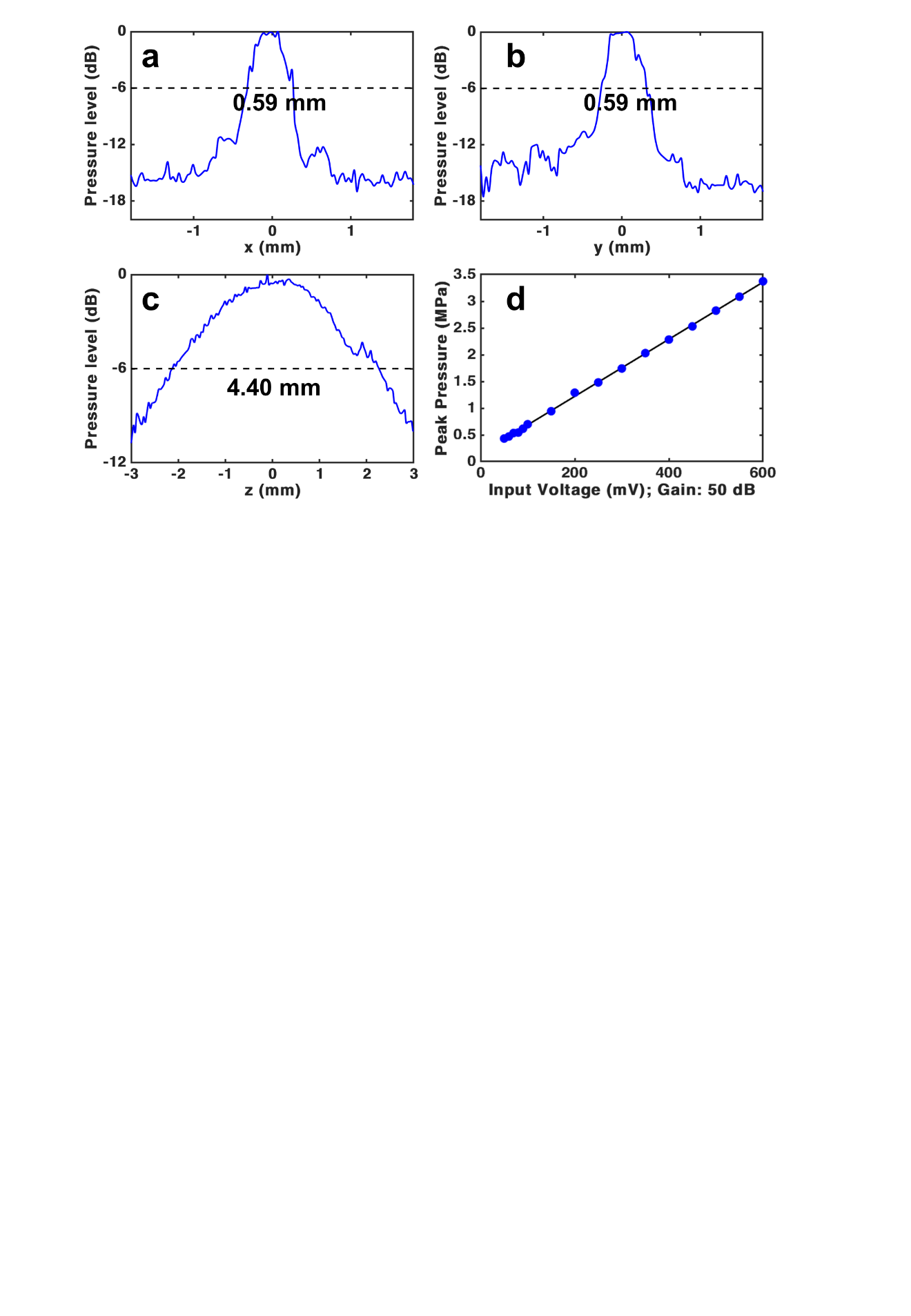
**

**Supplementary Figure 2:** **Free-space US field and pressure measured in hydrophone test. (a)** US field distribution and FWHM in x-direction. **(b)** US field distribution and FWHM in y-direction. **(c)** US field distribution and FWHM in z-direction. **(d)** Measured US pressures change along with the input voltage from the function generator.


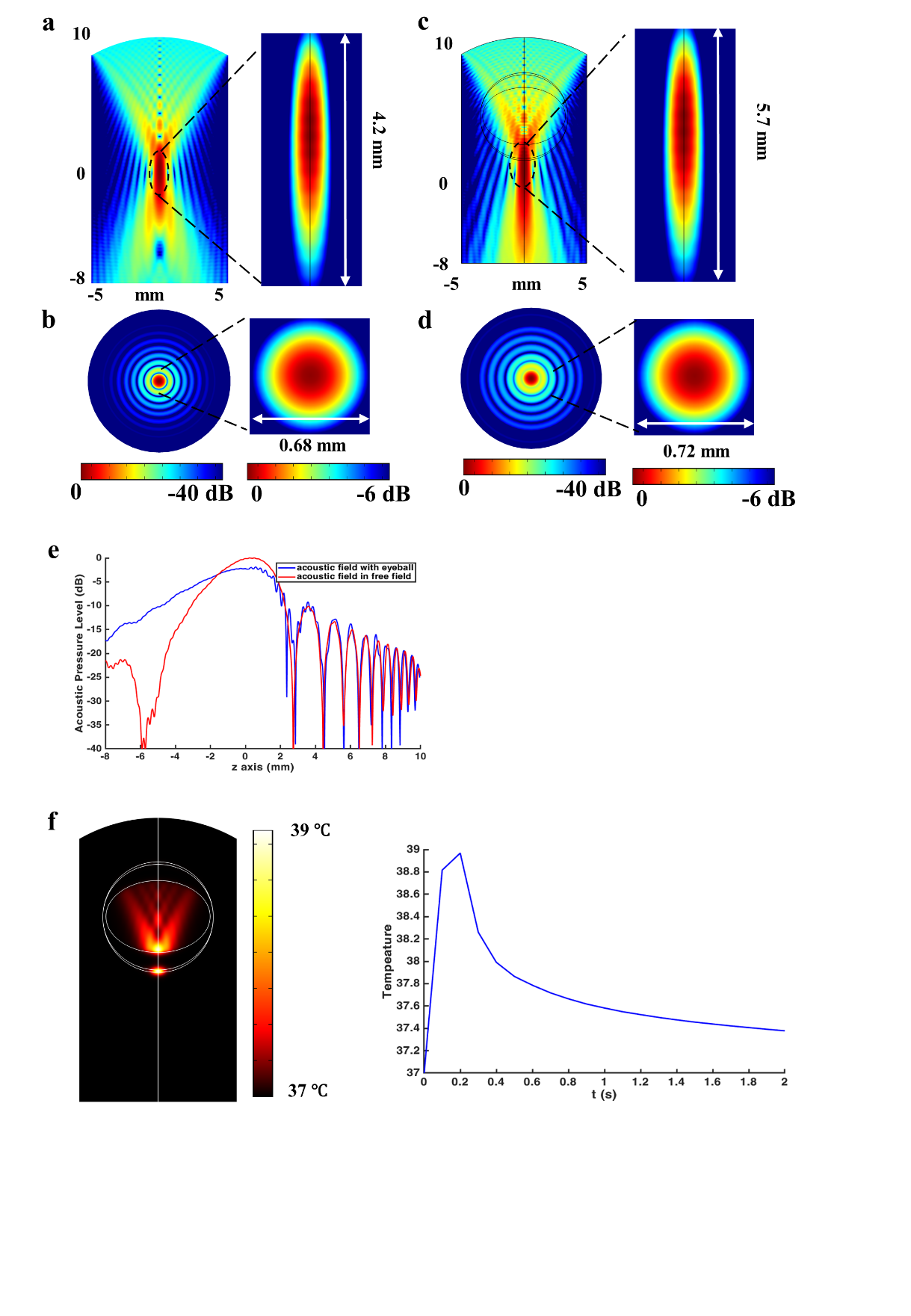


**Supplementary Figure 3: Simulated results of US distortions and attenuation caused by the eyeball. (a)** Simulated free-space US field in x-z plane. (**b)** Simulated free-space US field in x-y plane. (**c)** Simulated US field in x-z plane with the eyeball. (**d)** Simulated free-space US field in x-y plane with the eyeball. (**e)** US field distribution along the center z-axis. (**f)** US-induced temperature increases within the eyeball. Simulation US pressure was 2.28 MPa and US duration was 200 ms continuous waves.


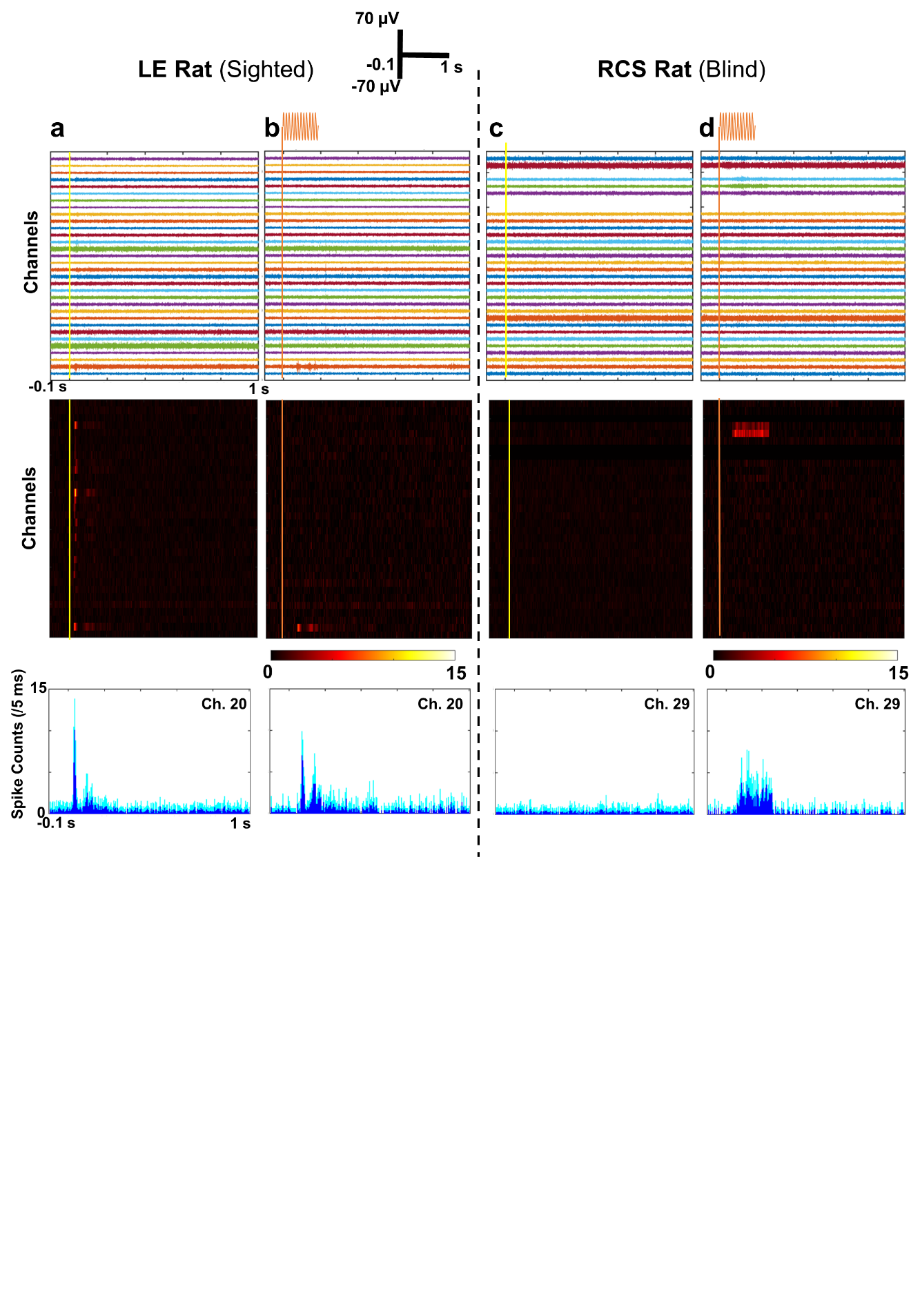


**Supplementary Figure 4: Examples of the evoked neuron activities recorded from VC *in vivo*. (a)** Light response from normal sighted rat. (**b)** US response from normal sighted rat. (**c)** Light response from blind rat. (**d)** US response from blind rat. The first row shows filtered signals by bandpass filter between 300 Hz and 7000 Hz with a scale of ± 70 µV in a time range from -0.1s to 1s. The second row shows the average spike counts per 5 ms of all channels. In the third row, a representative channel was randomly selected to demonstrate the spike counts curve with 12 repeated trials. The deep blue shows the average value, and the baby blue shows the standard deviation.

**
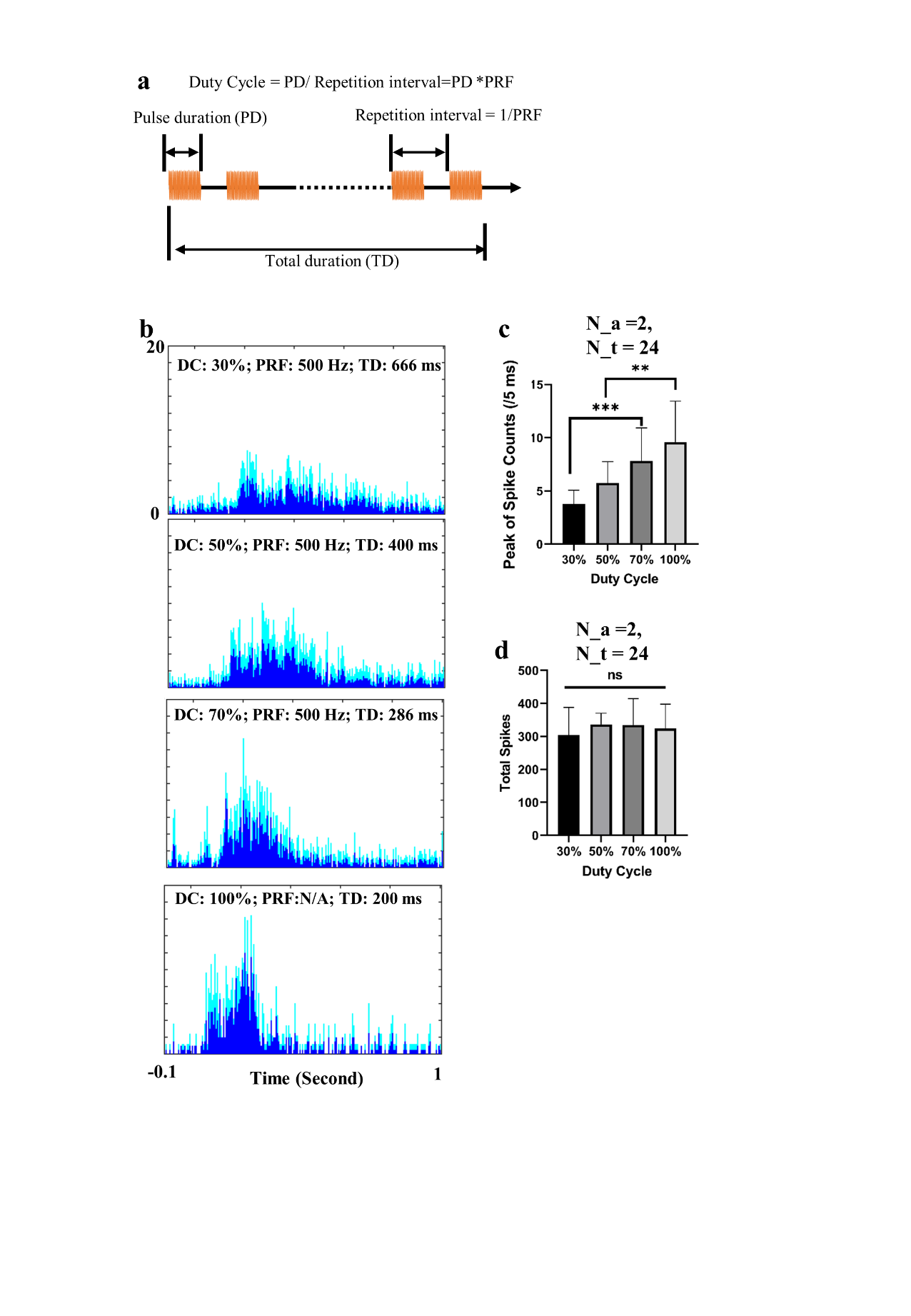
**

**Supplementary Figure 5:** **The US stimulation response determined by duty cycle.** (**a)** Schematic diagram of pulsed US sequence and US parameters. (**b)** Responses induced by US sequences with different DC and TD, while the effective US durations are kept the same for all cases. (**c)** The relationship between response amplitudes and DC. (**d)** The relationship between the number of total neuron spikes and DC.


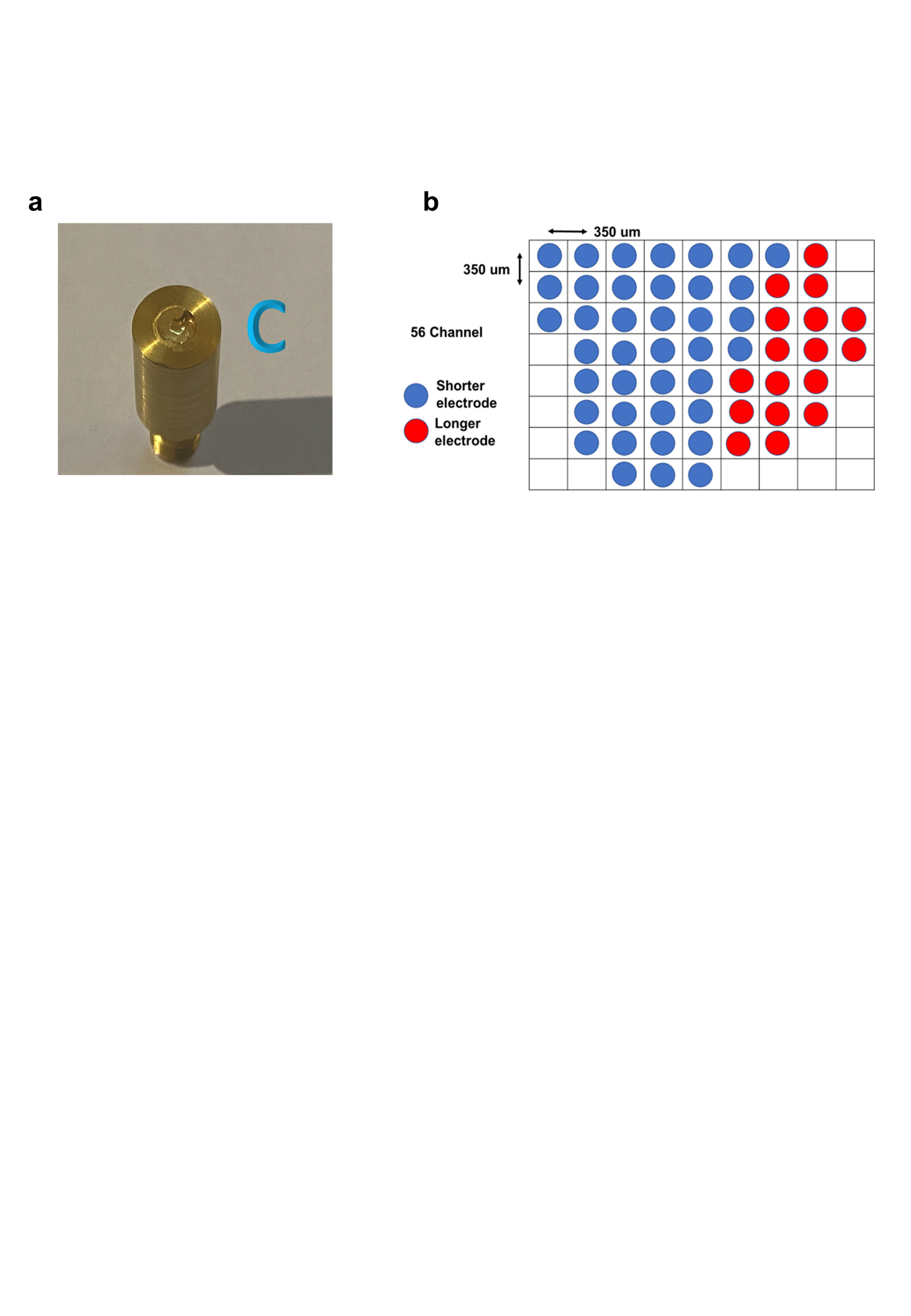


**Supplementary Figure 6:** **The helical transducer for pattern generation of letter form- ‘C’.** (**a)** The photography of the fabricated helical transducer. (**b)** The schematic diagram of the 56-channel MEA. To ensure the MEA record from the surface of SC, red electrodes are designed to be 0.1 mm longer than the blue electrodes, conforming with the curve of SC surface.

**
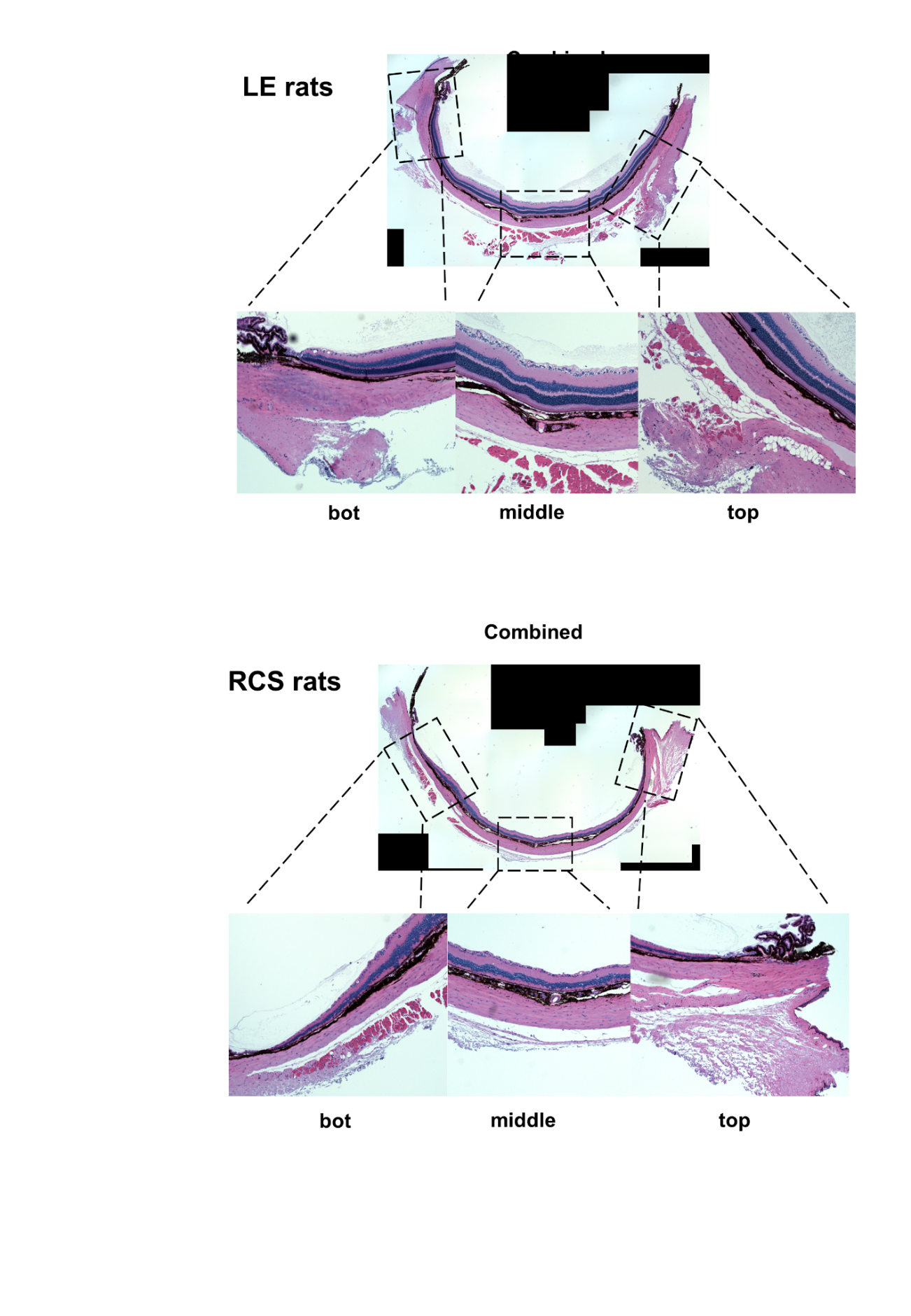
**

**Supplementary Figure 7:** **Representative histology results.** Histology results of US stimulated retinas from a normal sighted LE rat and a retinal degenerate blind RCS rat. Hematoxylin and eosin (H&E) stained images of the retina representing three different regions of the eye (superior, inferior and central) were microscopically examined. LE rats having normal retina showed no apparent damages after US stimulation. Besides the absence of a clear photoreceptor layer, no changes were observed in the retina of US stimulated RCS rat.


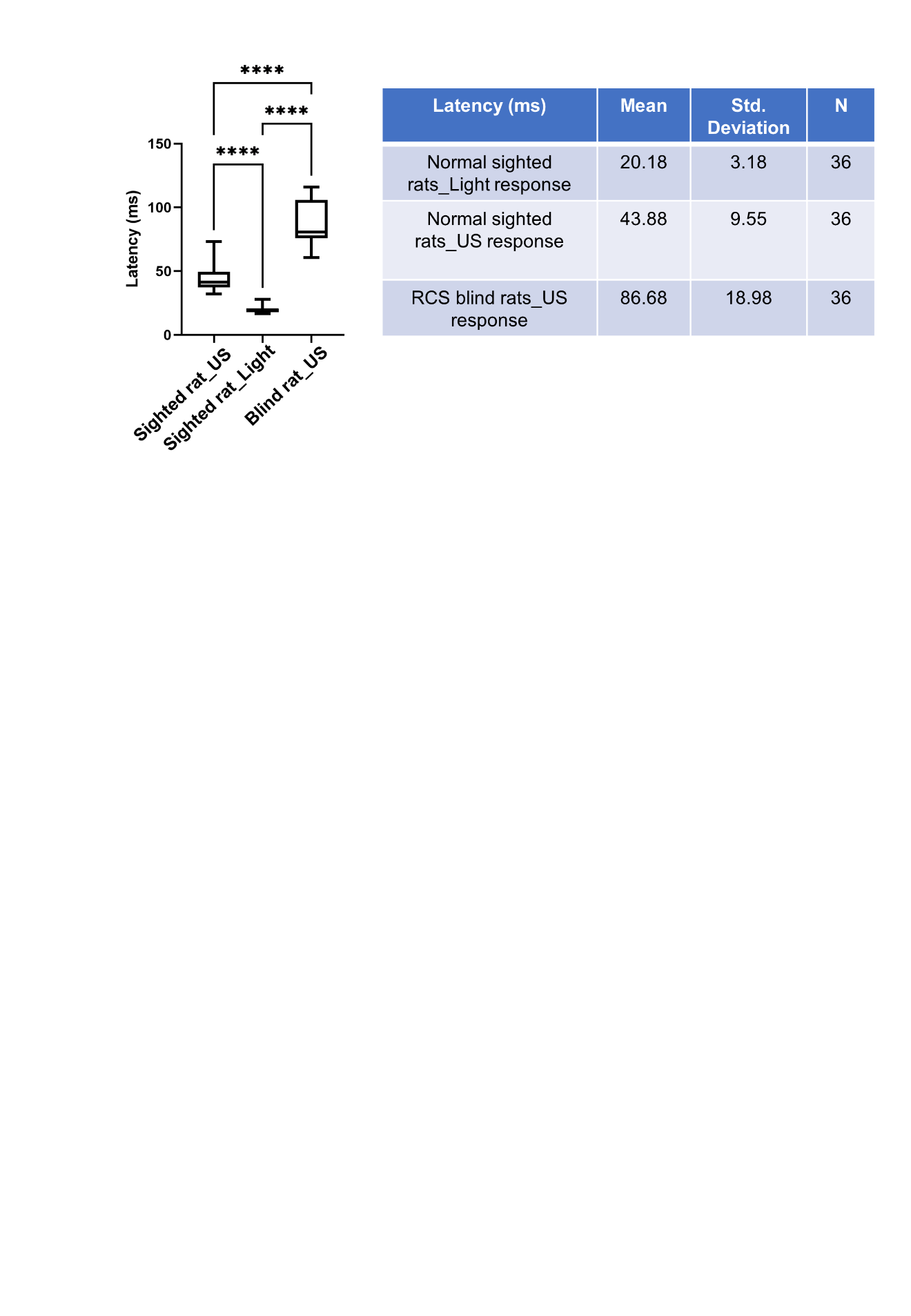


**Supplementary Figure 8:** Differences in the response latencies from both stimulation methods and both rat strains.
