# Supplementary material for: Noninvasive Ultrasound Retinal Stimulation for Vision Restoration at High Spatiotemporal Resolution
Source: BME Front. 2022 Feb 21;2022:9829316. doi: 10.34133/2022/9829316 (PMC10521738; doi:10.34133/2022/9829316)
Supplement: Supplementary Materials — Figure S1: the schematic diagram of the US sequence and the definition of US parameters in our study. Figure S2: free-space US field and pressure measured in the hydrophone test. Figure S3: simulated results of US distortions and attenuation caused by the eyeball. Figure S4: examples of US-evoked neuron activities recorded from VC. Figure S5: the US stimulation response determined by duty cycle. Figure S6: the helical transducer for pattern generation of the letter form “C”. Figure S7: representative histology results. Figure S8: differences in the response latencies from both stimulation methods and both rat strains. Table S1: the number of rats used in each subset of our study. Table S2: the relationship between the driving voltage of the US transducer and acoustic parameters. Table S3: list of acoustic and thermal parameters of water and ocular tissue components. [file 9829316.f1.zip › Table S1-S3.docx]

|  | **SC recording** | | | | **VC recording** | **Total** |
| --- | --- | --- | --- | --- | --- | --- |
|  | **Base** | **Spatial resolution** | **Temporal resolution** | **Change US parameters** |  |  |
| **Normal sighted rats** | **3** | **0** | **0** | **5** | **2** | **10** |
| **RCS blind rats** | **3** | **3** | **3** | **5** | **2** | **16** |

**Supplementary Table 1: The number of rats used in each subset of our study.** ‘Base’ represents that the rat was performed with light stimulation followed by ultrasound stimulation.

| Input Voltage (mV) | Pressure (MPa) | Isppa(W/cm^2) | MI |
| --- | --- | --- | --- |
| 50 | 0.432 | 6.220 | 0.245 |
| 70 | 0.535 | 9.535 | 0.304 |
| 100 | 0.699 | 16.306 | 0.397 |
| 150 | 0.943 | 29.639 | 0.536 |
| 200 | 1.289 | 55.416 | 0.732 |
| 250 | 1.481 | 73.121 | 0.841 |
| 300 | 1.742 | 101.144 | 0.989 |
| 350 | 2.030 | 137.353 | 1.153 |
| 400 | 2.283 | 173.790 | 1.297 |
| 450 | 2.530 | 213.398 | 1.437 |
| 500 | 2.825 | 265.979 | 1.604 |
| 550 | 3.086 | 317.368 | 1.753 |
| 600 | 3.374 | 379.374 | 1.916 |

**Supplementary Table 2:** The relationship between the driving voltage of US transducer (before 50 dB gain via power amplifier) and acoustic parameters such as negative peak pressure, intensity and mechanical index.

|  | Density (kg/m3) | Sound speed (m/s) | Heat capacity at constant pressure (J/kg/K) | Thermal conductivity (W/m/K) | Attenuation (dB/cm/MHz) |
| --- | --- | --- | --- | --- | --- |
| Water | 1000 | 1500 | 4178 | 0.62 | 0 |
| Cornea | 1062 | 1586 | 4178 | 0.58 | 0.78 |
| Vitreous | 1005 | 1532 | 3999 | 0.6 | 0.01 |
| Lens | 1076 | 1647 | 3000 | 0.40 | 1.19 |
| Retina | 1034 | 1538 | 3680 | 0.57 | 1.15 |

**Supplementary Table 3:** List of acoustic and thermal parameters of water and ocular tissue components.
