# Supplementary material for: Noninvasive Ultrasound Retinal Stimulation for Vision Restoration at High Spatiotemporal Resolution
Source: BME Front. 2022 Feb 21;2022:9829316. doi: 10.34133/2022/9829316 (PMC10521738; doi:10.34133/2022/9829316)
Supplement: Supplementary Materials — Figure S1: the schematic diagram of the US sequence and the definition of US parameters in our study. Figure S2: free-space US field and pressure measured in the hydrophone test. Figure S3: simulated results of US distortions and attenuation caused by the eyeball. Figure S4: examples of US-evoked neuron activities recorded from VC. Figure S5: the US stimulation response determined by duty cycle. Figure S6: the helical transducer for pattern generation of the letter form “C”. Figure S7: representative histology results. Figure S8: differences in the response latencies from both stimulation methods and both rat strains. Table S1: the number of rats used in each subset of our study. Table S2: the relationship between the driving voltage of the US transducer and acoustic parameters. Table S3: list of acoustic and thermal parameters of water and ocular tissue components. [file 9829316.f1.zip › renamed_c6565.pptx]

## Slide 1
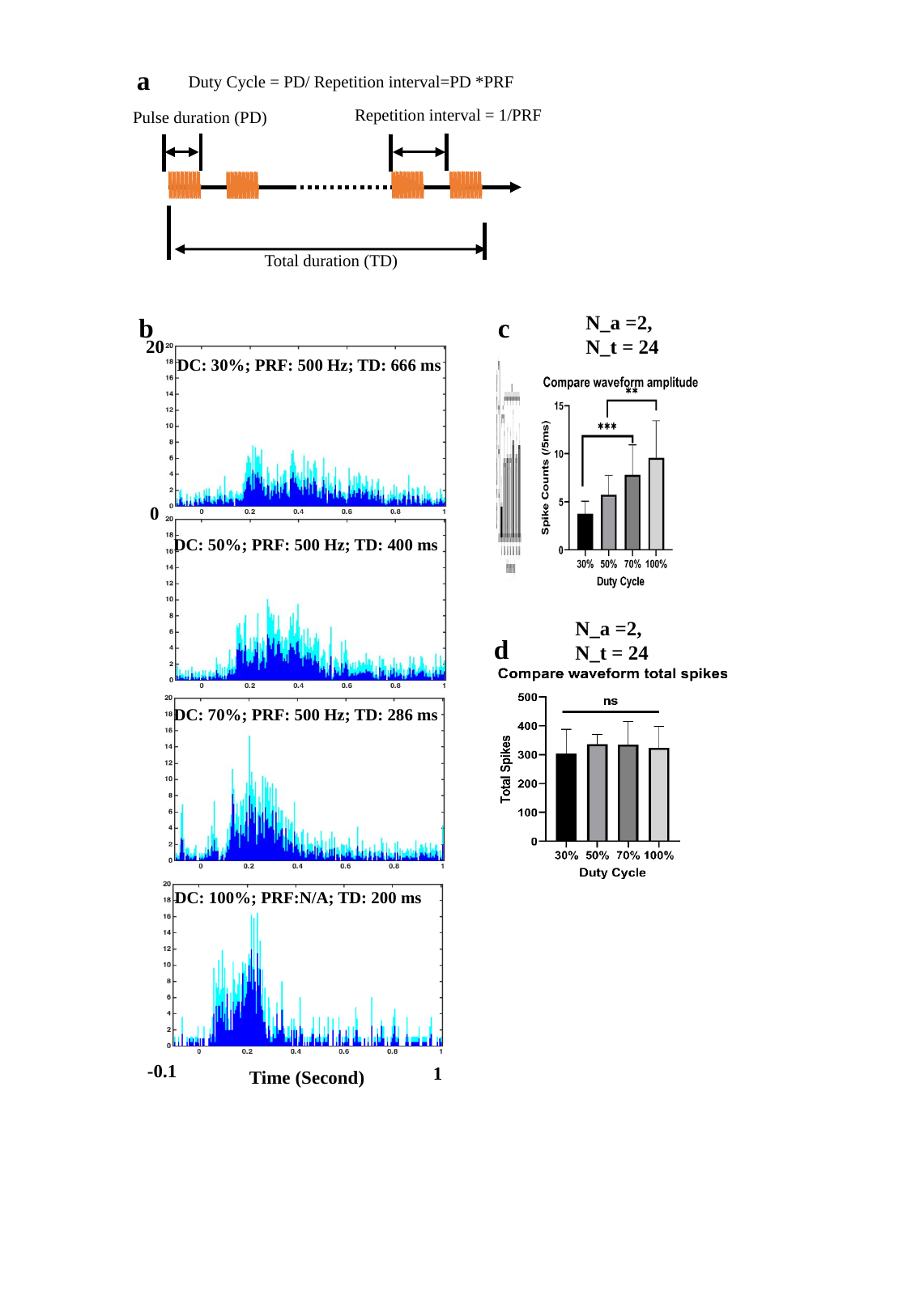

a
Duty Cycle = PD/ Repetition interval=PD *PRF
Repetition interval = 1/PRF
Pulse duration (PD)
Total duration (TD)
N_a =2,
N_t = 24
b
c
20
DC: 30%; PRF: 500 Hz; TD: 666 ms
0
DC: 50%; PRF: 500 Hz; TD: 400 ms
N_a =2,
N_t = 24
d
DC: 70%; PRF: 500 Hz; TD: 286 ms
DC: 100%; PRF:N/A; TD: 200 ms
-0.1
1
Time (Second)
