# Supplementary material for: Noninvasive Ultrasound Retinal Stimulation for Vision Restoration at High Spatiotemporal Resolution
Source: BME Front. 2022 Feb 21;2022:9829316. doi: 10.34133/2022/9829316 (PMC10521738; doi:10.34133/2022/9829316)
Supplement: Supplementary Materials — Figure S1: the schematic diagram of the US sequence and the definition of US parameters in our study. Figure S2: free-space US field and pressure measured in the hydrophone test. Figure S3: simulated results of US distortions and attenuation caused by the eyeball. Figure S4: examples of US-evoked neuron activities recorded from VC. Figure S5: the US stimulation response determined by duty cycle. Figure S6: the helical transducer for pattern generation of the letter form “C”. Figure S7: representative histology results. Figure S8: differences in the response latencies from both stimulation methods and both rat strains. Table S1: the number of rats used in each subset of our study. Table S2: the relationship between the driving voltage of the US transducer and acoustic parameters. Table S3: list of acoustic and thermal parameters of water and ocular tissue components. [file 9829316.f1.zip › renamed_71d8e.pptx]

## Slide 1
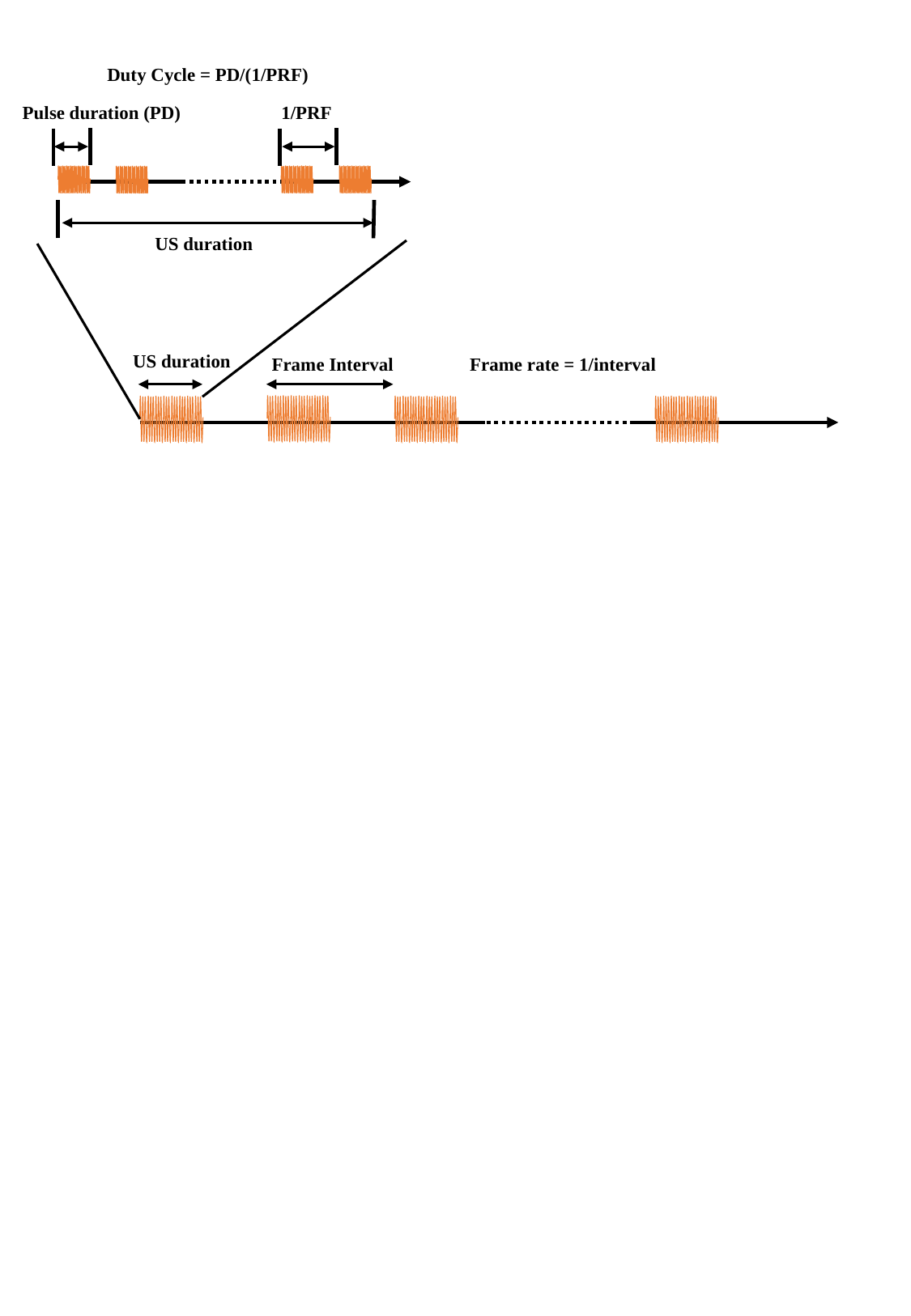

Duty Cycle = PD/(1/PRF)
Pulse duration (PD)
1/PRF
US duration
US duration
Frame Interval
Frame rate = 1/interval
