# Supplementary figures and images for: Noninvasive Ultrasound Retinal Stimulation for Vision Restoration at High Spatiotemporal Resolution
Source: BME Front. 2022 Feb 21;2022:9829316. doi: 10.34133/2022/9829316 (PMC10521738; doi:10.34133/2022/9829316)

## Slide 1
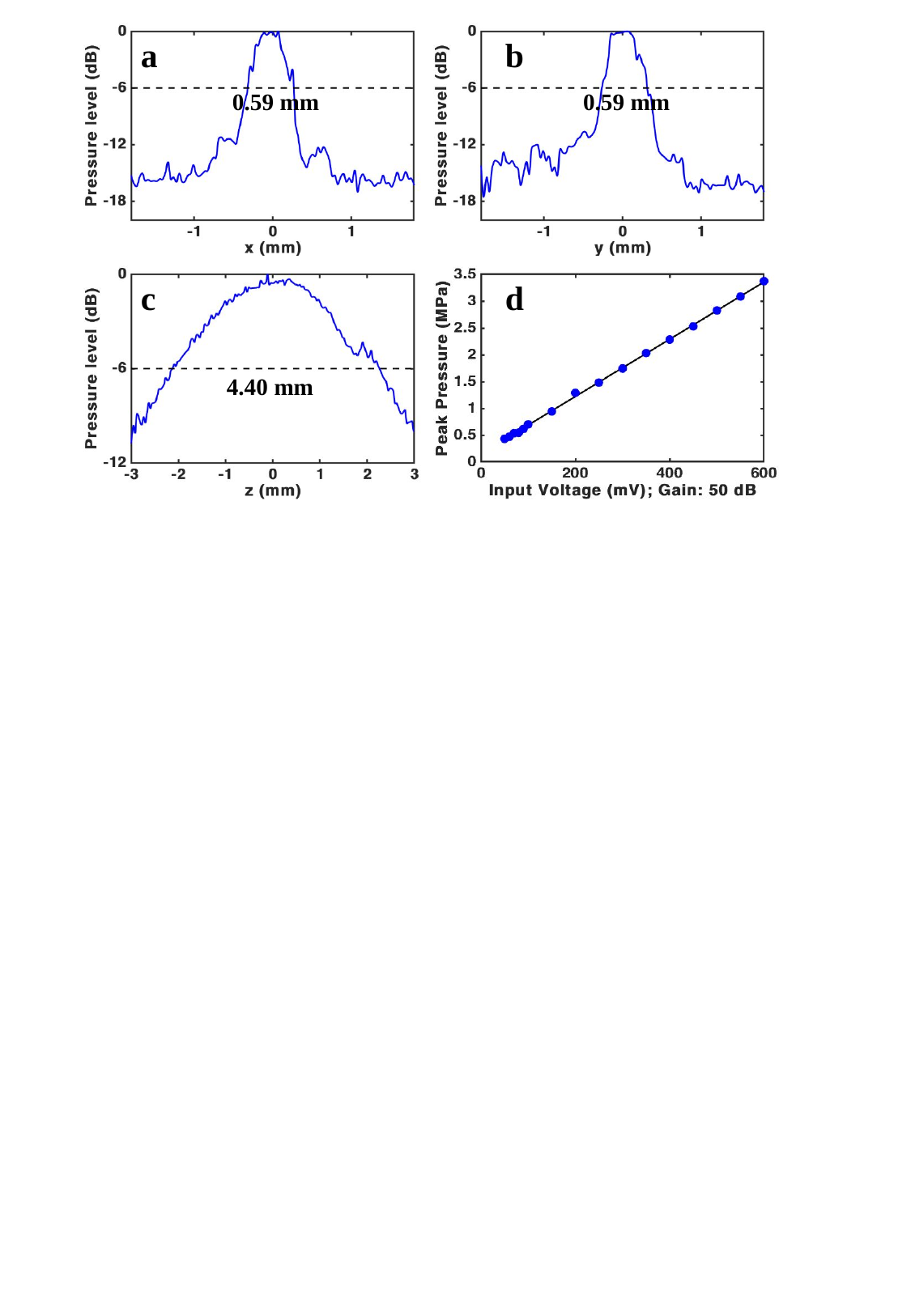

a
b
0.59 mm
0.59 mm
c
d
4.40 mm

Supplement: Supplementary Materials — Figure S1: the schematic diagram of the US sequence and the definition of US parameters in our study. Figure S2: free-space US field and pressure measured in the hydrophone test. Figure S3: simulated results of US distortions and attenuation caused by the eyeball. Figure S4: examples of US-evoked neuron activities recorded from VC. Figure S5: the US stimulation response determined by duty cycle. Figure S6: the helical transducer for pattern generation of the letter form “C”. Figure S7: representative histology results. Figure S8: differences in the response latencies from both stimulation methods and both rat strains. Table S1: the number of rats used in each subset of our study. Table S2: the relationship between the driving voltage of the US transducer and acoustic parameters. Table S3: list of acoustic and thermal parameters of water and ocular tissue components. [file 9829316.f1.zip › renamed_ec016.pptx]

## Slide 1
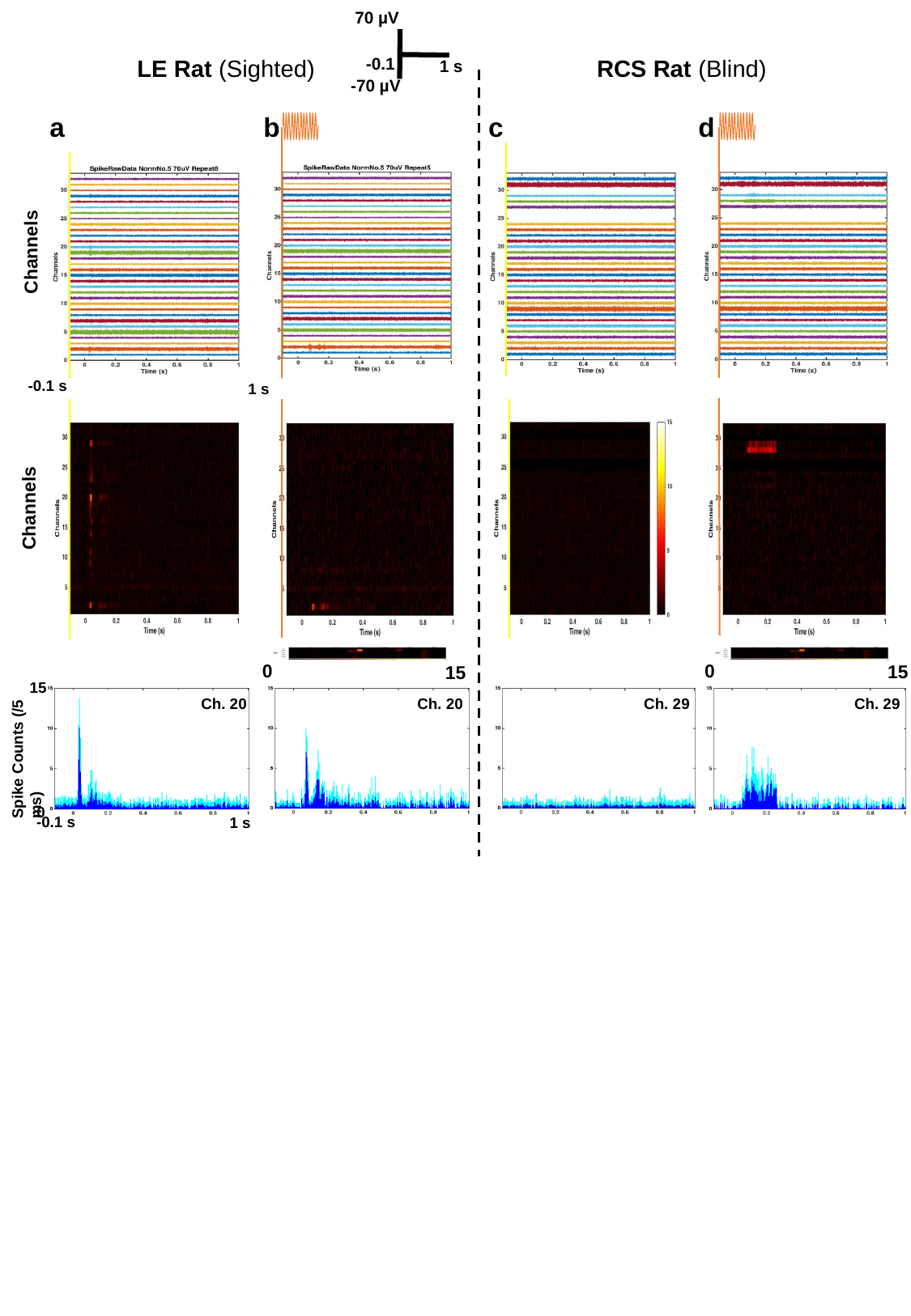

70 µV
-0.1
1 s
-70 µV
LE Rat (Sighted)
RCS Rat (Blind)
a
b
c
d
Channels
-0.1 s
1 s
Channels
0
15
0
15
15
Ch. 20
Ch. 20
Ch. 29
Ch. 29
Spike Counts (/5 ms)
0
-0.1 s
1 s

Supplement: Supplementary Materials — Figure S1: the schematic diagram of the US sequence and the definition of US parameters in our study. Figure S2: free-space US field and pressure measured in the hydrophone test. Figure S3: simulated results of US distortions and attenuation caused by the eyeball. Figure S4: examples of US-evoked neuron activities recorded from VC. Figure S5: the US stimulation response determined by duty cycle. Figure S6: the helical transducer for pattern generation of the letter form “C”. Figure S7: representative histology results. Figure S8: differences in the response latencies from both stimulation methods and both rat strains. Table S1: the number of rats used in each subset of our study. Table S2: the relationship between the driving voltage of the US transducer and acoustic parameters. Table S3: list of acoustic and thermal parameters of water and ocular tissue components. [file 9829316.f1.zip › renamed_87223.pptx]

## Slide 1
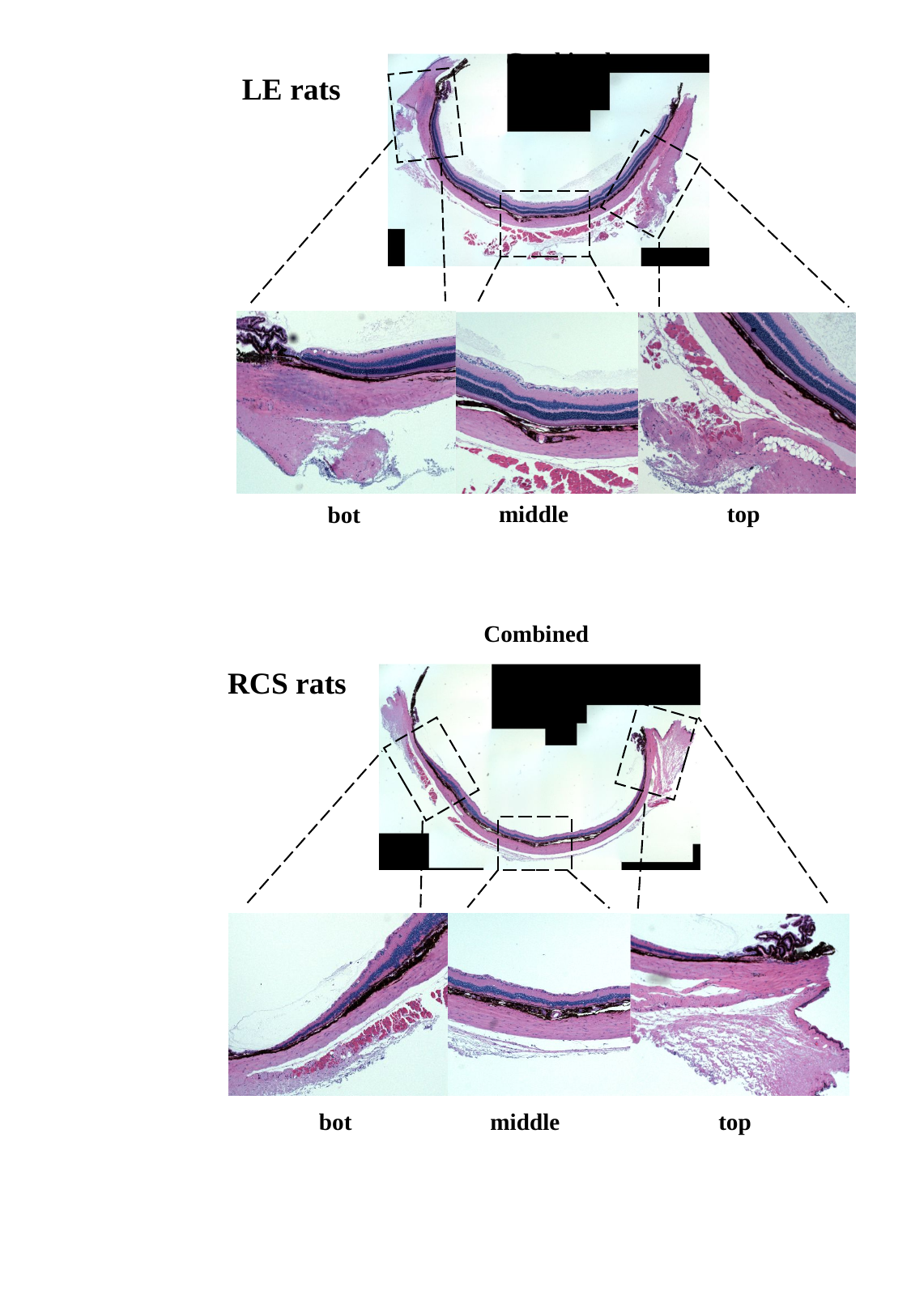

Combined
LE rats
middle
top
bot
Combined
RCS rats
middle
top
bot

Supplement: Supplementary Materials — Figure S1: the schematic diagram of the US sequence and the definition of US parameters in our study. Figure S2: free-space US field and pressure measured in the hydrophone test. Figure S3: simulated results of US distortions and attenuation caused by the eyeball. Figure S4: examples of US-evoked neuron activities recorded from VC. Figure S5: the US stimulation response determined by duty cycle. Figure S6: the helical transducer for pattern generation of the letter form “C”. Figure S7: representative histology results. Figure S8: differences in the response latencies from both stimulation methods and both rat strains. Table S1: the number of rats used in each subset of our study. Table S2: the relationship between the driving voltage of the US transducer and acoustic parameters. Table S3: list of acoustic and thermal parameters of water and ocular tissue components. [file 9829316.f1.zip › renamed_8148f.pptx]

## Slide 1
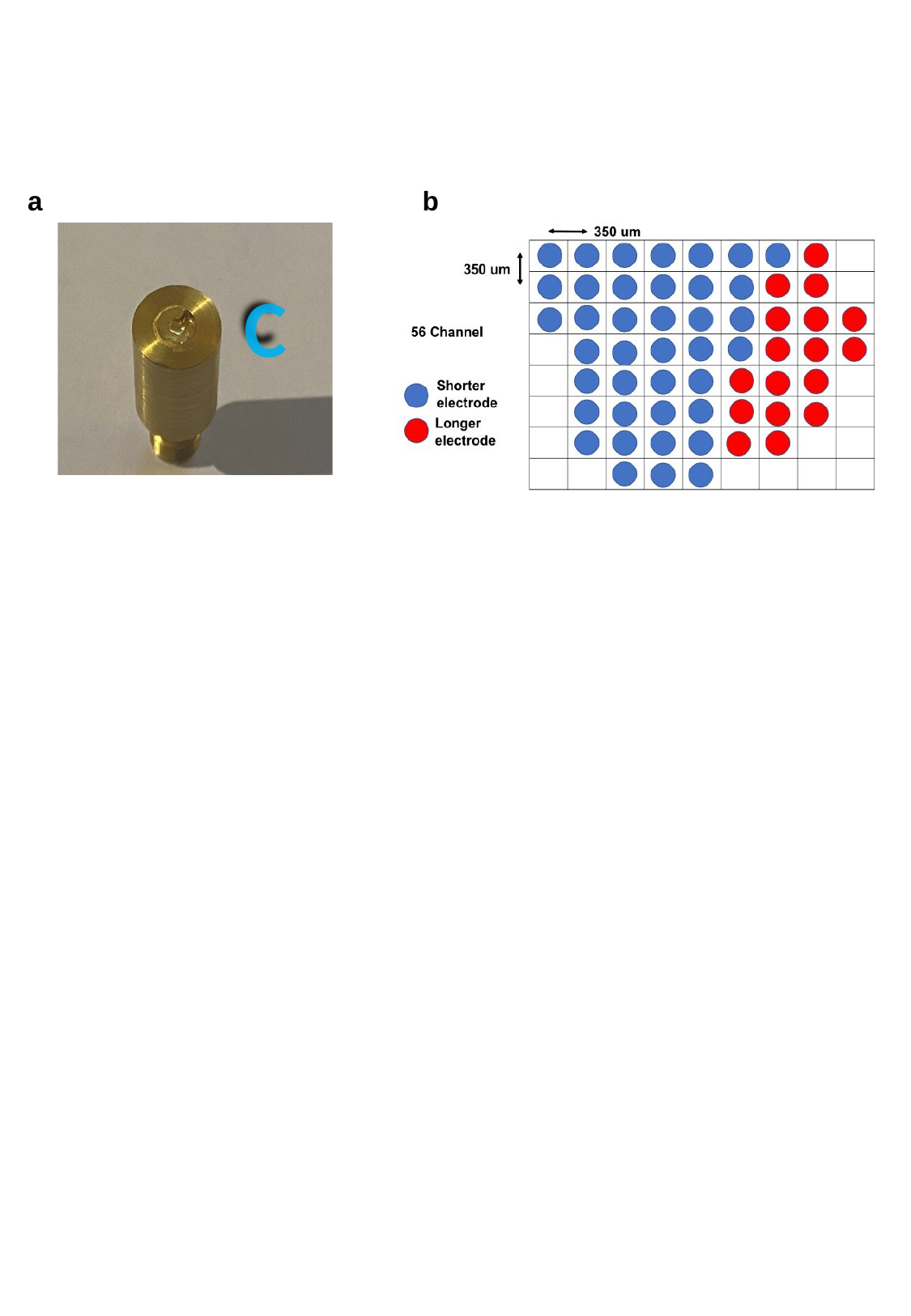

a
b
C

Supplement: Supplementary Materials — Figure S1: the schematic diagram of the US sequence and the definition of US parameters in our study. Figure S2: free-space US field and pressure measured in the hydrophone test. Figure S3: simulated results of US distortions and attenuation caused by the eyeball. Figure S4: examples of US-evoked neuron activities recorded from VC. Figure S5: the US stimulation response determined by duty cycle. Figure S6: the helical transducer for pattern generation of the letter form “C”. Figure S7: representative histology results. Figure S8: differences in the response latencies from both stimulation methods and both rat strains. Table S1: the number of rats used in each subset of our study. Table S2: the relationship between the driving voltage of the US transducer and acoustic parameters. Table S3: list of acoustic and thermal parameters of water and ocular tissue components. [file 9829316.f1.zip › renamed_8dc1b.pptx]

## Slide 1
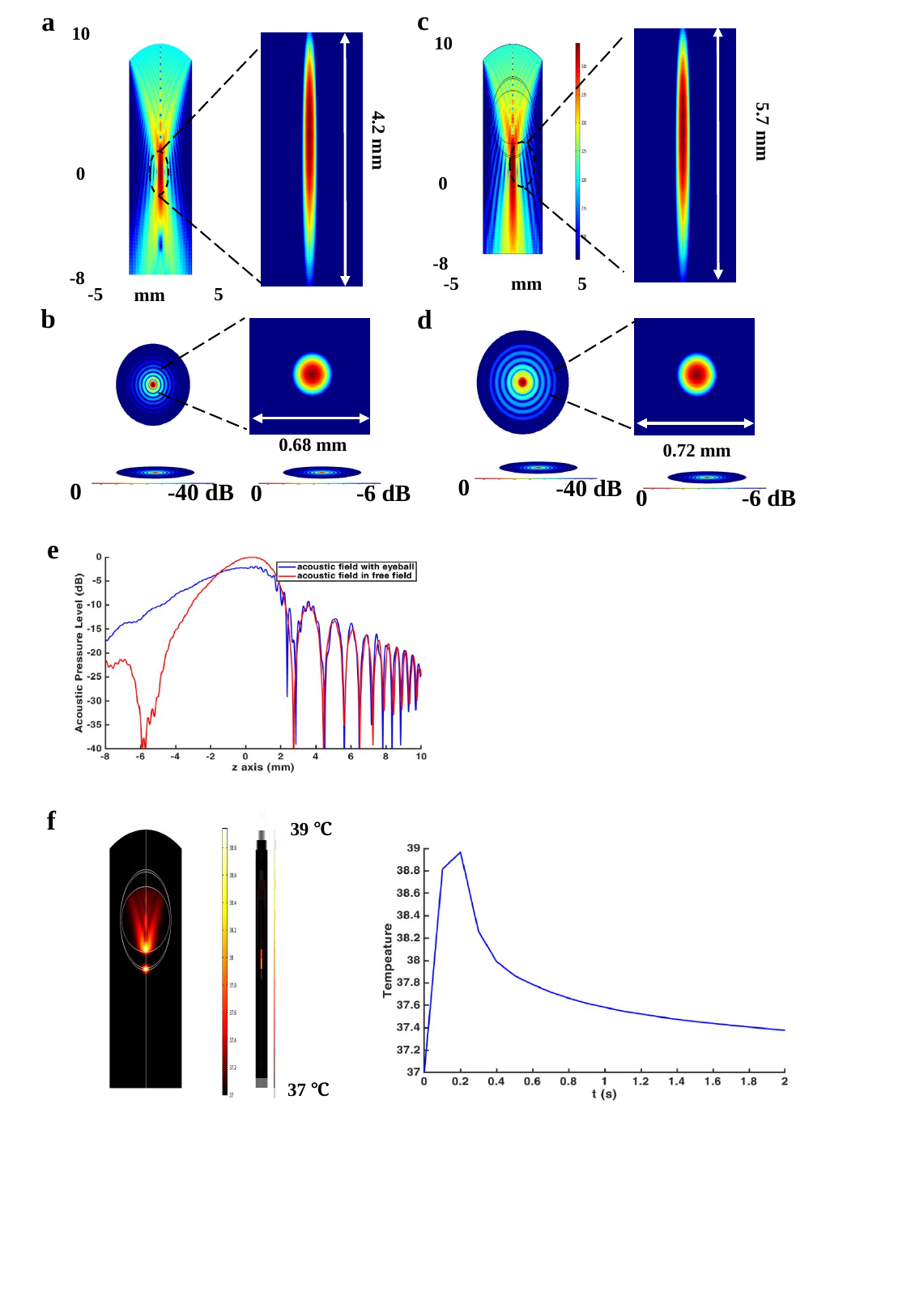

c
a
10
4.2 mm
0
-8
-5
5
mm
b
0.68 mm
0
-40 dB
0
-6 dB
10
5.7 mm
0
-8
-5
mm
5
d
0
-40 dB
0.72 mm
0
-6 dB
e
f
39 ℃
37 ℃

Supplement: Supplementary Materials — Figure S1: the schematic diagram of the US sequence and the definition of US parameters in our study. Figure S2: free-space US field and pressure measured in the hydrophone test. Figure S3: simulated results of US distortions and attenuation caused by the eyeball. Figure S4: examples of US-evoked neuron activities recorded from VC. Figure S5: the US stimulation response determined by duty cycle. Figure S6: the helical transducer for pattern generation of the letter form “C”. Figure S7: representative histology results. Figure S8: differences in the response latencies from both stimulation methods and both rat strains. Table S1: the number of rats used in each subset of our study. Table S2: the relationship between the driving voltage of the US transducer and acoustic parameters. Table S3: list of acoustic and thermal parameters of water and ocular tissue components. [file 9829316.f1.zip › renamed_5b4a4.pptx]
